# Design, Synthesis, Antimycobacterial Evaluation, and In Silico Studies of 3-(Phenylcarbamoyl)-pyrazine-2-carboxylic Acids

**DOI:** 10.3390/molecules22091491

**Published:** 2017-09-07

**Authors:** Lucia Semelková, Petra Janošcová, Carlos Fernandes, Ghada Bouz, Ondřej Janďourek, Klára Konečná, Pavla Paterová, Lucie Navrátilová, Jiří Kuneš, Martin Doležal, Jan Zitko

**Affiliations:** 1 Faculty of Pharmacy in Hradec Králové, Charles University, Heyrovského 1203, Hradec Králové 500 05, Czech Republic; Petra.Janoscova@seznam.cz (P.J.); oaksdonsad@gmail.com (C.F.); bouzg@faf.cuni.cz (G.B.); JANDO6AA@faf.cuni.cz (O.J.); konecna@faf.cuni.cz (K.K.); navratl2@faf.cuni.cz (L.N.); kunes@faf.cuni.cz (J.K.); dolezalm@faf.cuni.cz (M.D.); 2 Department of Clinical Microbiology, University Hospital, Sokolská 581, Hradec Králové 500 05, Czech Republic; pavla.paterova@fnhk.cz

**Keywords:** anilides, antimycobacterial activity, cytotoxicity, DprE1, pyrazinamide, pyrazinoic acid, RpsA

## Abstract

Pyrazinamide, the first-line antitubercular drug, has been regarded the basic component of tuberculosis treatment for over sixty years. Researchers have investigated its effect on *Mycobacterium tuberculosis* for this long time, and as a result, new potential targets of pyrazinamide or its active form, pyrazinoic acid, have been found. We have designed and prepared 3-(phenyl-carbamoyl)pyrazine-2-carboxylic acids as more lipophilic derivatives of pyrazinoic acid. We also prepared methyl and propyl derivatives as prodrugs with further increased lipophilicity. Antimycobacterial, antibacterial and antifungal growth inhibiting activity was investigated in all prepared compounds. 3-[(4-Nitrophenyl)carbamoyl]pyrazine-2-carboxylic acid (**16**) exerted high antimycobacterial activity against *Mycobacterium tuberculosis* H37Rv with MIC = 1.56 μg·mL^−1^ (5 μM). Propyl 3-{[4-(trifluoromethyl)phenyl]carbamoyl}pyrazine-2-carboxylate (**18a**) showed also high antimycobacterial activity against *Mycobacterium tuberculosis* H37Rv with MIC = 3.13 μg·mL^−1^. In vitro cytotoxicity of the active compounds was investigated and no significant cytotoxic effect was observed. Based to structural similarity to known inhibitors of decaprenylphosphoryl-β-d-ribose oxidase, DprE1, we performed molecular docking of the prepared acids to DprE1. These in silico experiments indicate that modification of the linker connecting aromatic parts of molecule does not have any negative influence on the binding.

## 1. Introduction

**Figure 1:**
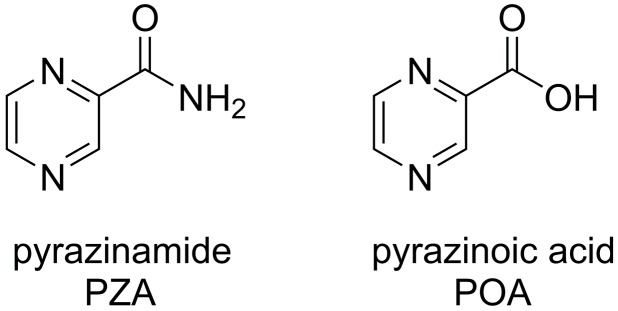
Structures of pyrazinamide and its active form, pyrazinoic acid.

Pyrazinamide (PZA, Figure 1) is one of the fist-line drugs used in the treatment of tuberculosis (TB). Due to its unique sterilizing effect on semi-dormant tubercle bacilli and ability to shorten the duration of therapy, PZA has been used for over sixty years in combination with other antituberculotics [1,2,3]. Although PZA has been applied for such a long time, the mechanism of action is still not yet completely understood. PZA is known to be a prodrug that is converted to its active form, pyrazinoic acid (POA), by the enzyme pyrazinamidase/nicotinamidase inside the mycobacterial cell [4].

Its non-specific mechanism of action is based on a cyclic expulsion of POA anion to the extracellular space, protonation and penetration of the uncharged POA back to the cell. This cycle results in proton accumulation and potentially to cytoplasmic acidification along with the possible collapse of membrane potential and membrane transport [5,6]. With respect to previous observations and the p*K*_a_ of POA, an acidic pH is considered important for the PZA/POA activity. However, recent experiments by Peterson et al. [7] disputed the acidification of cytoplasm as a mechanism of action of PZA and POA and showed that the acidification of intracellular compartment required PZA/POA concentrations at least 1–2 orders higher than minimum inhibitory concentrations (MICs). Specific targets of PZA/POA were suggested as Fatty Acid Synthase (FAS) I (involved in mycolic acid biosynthesis) [8], aspartate decarboxylase (biosynthetic pathway of coenzyme A) [9,10] and ribosomal protein S1 (RpsA, important for trans-translation) [11].

RpsA, a 30S ribosomal protein S1, is essential for translation and is involved in trans-translation, which is the process of rescuing ribosomes stalled in the translation of mRNA [12]. Shi et al. [11] proposed that POA binds directly to the C-terminus region and disrupts the formation of RpsA-tmRNA complex. Experiments of Yang et al. [13] have pointed to the fourth S1 domain of RpsA as the binding site for POA, where the surface is supposed to interact with tmRNA [13,14].

POA itself cannot penetrate through the lipid-rich mycobacterial cell wall easily due to its low lipophilicity and significant acidity (p*K*_a_ = 2.9) [5] According to the performed experiments, Yang et al. [13] suggested a substitution with any lipophilic moiety in the position 5 or 6 on the pyrazine ring. This hypothesis is based on the interactions observed in a co-crystallized complex of POA-RpsA, where the substituents at C-5 and C-6 of the pyrazine ring seem to be less critical for POA binding to S1 domain.

Our research group has focused on the synthesis of *N*-phenylpyrazine-2-carboxamides for several years. Many of these compounds exerted antimycobacterial effect comparable to PZA (MIC = 6.25–12.5 μg·mL^−1^, Šula´s medium, pH = 5.6 [15,16,17]). Considering the previous results of *N*-phenylcarboxamides (anilides), we decided to choose the phenylcarbamoyl moiety as a lipophilic substituent for the synthesis of more lipophilic POA derivatives as Yang et al. have suggested. We intend to prepare derivatives substituted in positions 5, 6 and even 3 to study the influence of positional isomerism. First, we decided to prepare substituted derivatives of POA with phenylcarbamoyl substituent in position 3.

We thus prepared a series of 18 substituted POA derivatives and also 17 propyl esters and 12 methyl esters from the synthesized acids to increase their lipophilicity and possibly enhance the penetration through the lipid-rich mycobacterial cell wall. After synthesis of these derivatives, we went through literature and found an article of Neres et al. [18] describing 2-carboxyquinoxalines as inhibitors of mycobacterial decaprenylphosphoryl-β-d-ribose oxidase (DprE1). DprE1 is an essential enzyme involved in the biosynthesis of arabinogalactan, a basic component of mycobacterial cell wall [19,20]. Nitrobenzothiazinones have become the first effective inhibitors of DprE1 [21]. Since then, the modification and design of new potential inhibitors continue. Discovered inhibitors belong to many structural classes, namely azaindoles [22], 4-aminoquinolone piperidine amides [23], pyrazolopyridones [24], 8-pyrrolobenzothiazinones [25], benzothiazolylpyrimidine-5-carboxamides [26], and 2-carboxyquinoxalines [18], among others.

**Figure 2:**
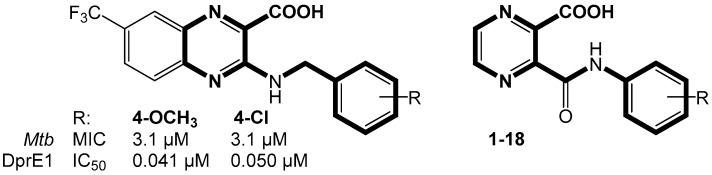
Comparison of the basic scaffold of previously prepared DprE1 inhibitors and structures presented in this paper.

Although the 3-substituted derivatives may fail to bind to the RpsA for the reasons mentioned above, their structural similarity to known inhibitors of DprE1 (Figure 2) led us to perform a molecular docking to DprE1. We intended to study the effect of structural differences between reported inhibitors and our compounds, that is, the absence of the second condensed ring and alteration of -NH-CH_2_- linker.

## 2. Results and Discussion

### 2.1. Chemistry

In this project 18 compounds **1**–**18** from a series of substituted 3-(phenylcarbamoyl)pyrazine-2-carboxylic acids, 17 related propyl esters **1a**–**18a** and 12 related methyl esters **1b**, **4b**–**6b**, **8b**, **11b**–**15b** and **17b**, **18b** were prepared. Compounds **1**, **7**, **9**, **11**, **16** and **18** were published previously as semi-products—see Section 3.2.3 for references.

**Scheme 1:**
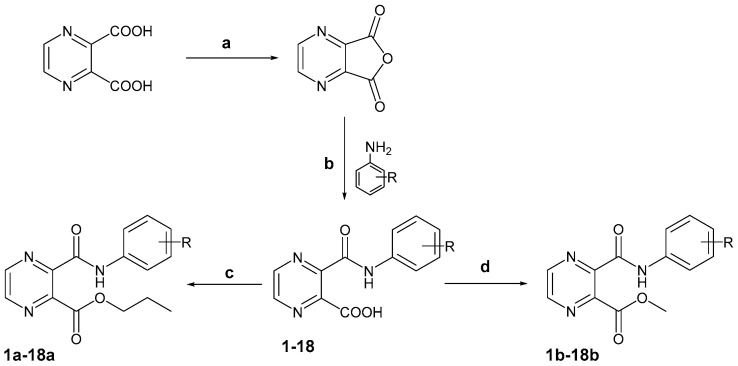
Synthetic procedures of prepared series. Reagents and Conditions: (**a**) acetic anhydride, reflux, 1 h; (**b**) 1. tetrahydrofuran, RT, 1 h, 2. water, NaHCO_3_ sol.; (**c**) propanol, H_2_SO_4_, MW: 120 °C, 20 min, 50 W; (**d**) methanol, H_2_SO_4_, MW: 120 °C, 20 min, 50 W.

The majority of compounds from the acid series were prepared starting from commercially available pyrazine-2,3-dicarboxylic anhydride and the synthetic procedure was performed according to [27] with some modifications, see Scheme 1. Several compounds were prepared using pyrazine-2,3-dicarboxylic acid as a starting compound. Pyrazine-2,3-dicarboxylic acid was treated in acetic acid anhydride at reflux to convert to pyrazine-2,3-dicarboxylic anhydride; the procedure was modified on the basis of the same literature. The analytical characteristics of pyrazine-2,3-dicarboxylic anhydride were in accordance with published literature data. Subsequently the anhydride was dissolved in tetrahydrofuran and a corresponding substituted aniline was added. The reaction mixture was stirred for 1 h at RT. Afterwards water was poured in and a saturated aqueous solution of NaHCO_3_ was added dropwise to pH 6 in order to form crystals of the desired substituted 3-(phenylcarbamoyl)pyrazine-2-carboxylic acids **1**–**18** in 58–98% yield. Propyl esters **1a**–**18a** were prepared via esterification in propanol with a catalytic amount of H_2_SO_4_ in 35–85% yield. Methyl esters **1b**–**18b** of several of the acids (**1**, **4**–**6**, **8**, **11**–**15** and **17**, **18**) were also prepared using the same procedure as propyl esters with methanol instead as the solvent in 52–76% yield. The esterification was carried out under microwave irradiation to accelerate the progress of the reaction. The reaction was performed under mild conditions in a microwave reactor to facilitate the forming of the ester bond and to prevent a decomposition of the carboxylic moiety. Despite the mild conditions and an effort to avoid decarboxylation, we have not been successful in the synthesis of propyl ester of compound **16** (the presence of the decarboxylation product was confirmed by NMR). Subsequently, synthesis of the ester **16a** was attempted by conversion of the acid **16** to its acyl chloride by conventional addition of SOCl_2_, followed by alcoholysis by propanol in the presence of pyridine as a base. However, this was not successful either, due to the low yields.

All compounds were characterized by ^1^H- and ^13^C-NMR, IR, elemental analysis, and melting point. The ^1^H-NMR spectra showed the proton peaks of the carboxylic groups in the range of 13.91–13.73 ppm. In case of some acids, this peak was not visible due to exchange with D_2_O. Peaks for the pyrazine hydrogens were in the range of 8.99–8.86 in DMSO-*d*_6_ and 8.82–8.68 in CDCl_3_. They were detected as one peak or as two peaks with coupling constant *J* ranging between 2.2–2.7 Hz, which is in accordance with the literature [28]. The IR spectroscopy confirmed the presence of the expected characteristic functional groups. The C=O stretching bands were located at 1697–1611 cm^−1^ for amidic moiety, 1757–1701 cm^−1^ for carboxylic acids, and 1748–1727 cm^−1^ for esters. Amidic N-H stretching bands were at 3379–3106 cm^−1^ and carboxylic O-H stretching at 3187–2584 cm^−1^. Esters showed an alkyl C-H stretching in the range of 2983–2919 cm^−1^.

The stability of the prepared esters in DMSO solution was studied. Samples were kept in the refrigerator (7 °C) for one month and subsequently the stability was verified by TLC (mobile phase: propanol/30% aq. sol. of ammonia 3:1) in comparison with the original acid. The temperature used for stability validation was based on storage conditions of dissolved samples before biological testing. No presence of the original acid was observed in the studied samples.

### 2.2. Lipophilicity

Lipophilicity is an important physico-chemical property of drugs or biologically active compounds. This property determines the penetration through biological membranes by passive diffusion. A successful biological effect depends on an appropriate ratio between the hydrophilic and hydrophobic properties of the substance. This aspect is very important especially for antitubercular drugs due to the presence of the lipid-rich mycobacterial wall. Log *P* values, mentioned in Table 1, were calculated by ChemBioDraw Ultra 14.0. The log *P* value of POA is −0.66. The average increase of lipophilicity of acids **1**–**18**, compared to POA, was 1.59 ± 0.58 (*n* = 18). Further esterification of **1**–**18** increased log *P* by 0.28 ± 0.06 (*n* = 12) in the case of methyl esters (**1b**, **4b**–**6b**, **8b**, **11b**–**15b, 17b, 18b**) and 1.09 ± 0.03 (*n* = 17) for propyl esters **1a**–**18a**.

### 2.3. Antimycobacterial Evaluation

Antimycobacterial in vitro screening was performed on four mycobacterial strains—*Mtb*, *M. avium*, *M. kansasii* and fast growing *M. smegmatis*—using a Microplate Alamar Blue Assay (MABA; see the Supplementary Material for the experimental details). The antimycobacterial activity results were expressed as the minimum inhibitory concentration (MIC) in μg·mL^−1^. Three standards were used—POA, PZA and isoniazid (INH).

Only six compounds from **1**–**18** exerted certain antimycobacterial activity against *Mtb*, the results are presented in Table 1. Compounds **10**, **14** and **17** showed activity comparable with PZA and POA (MIC = 100 μg·mL^−1^). The antimycobacterial activity of PZA and POA is dependent on the conditions of biological assays; the activity increases with a decrease of the environmental pH [29,30]. For example, in acidic pH = 5.6 PZA exerts MIC of 12.5–25 µg·mL^−1^ [16] yet is only weakly active at pH = 6 with MIC = 200 µg·mL^−1^ [30]. Compounds **2** and **11** showed moderate activity with MIC = 50 μg·mL^−1^. The most active compound from the series of acids was compound **16** with 4-NO_2_ substitution on phenyl ring with MIC = 1.56 μg·mL^−1^ (5 μM). Although this activity can be considered as high, it does not achieve the antimycobacterial activity of isoniazid (MIC = 0.1–0.2 μg·mL^−1^; 0.7–1.5 μM).

In general, the increase of lipophilicity via conversion of the acids to their propyl or methyl esters did not cause any significant improvement in antimycobacterial activity against *Mtb*. The only exception was compound **18a**, which displayed activity with MIC = 3.13 μg·mL^−1^ (10 μM). Compound **2** with 2-OH substitution kept its antimycobacterial effect after conversion to the propyl ester **2a** (MIC = 50 μg·mL^−1^). Methyl esters did not exert any antimycobacterial activity at the tested concentrations. None of the presented compounds proved any significant activity against *M. kansasii*, *M. avium* and *M. smegmatis*.

### 2.4. Antibacterial and Antifungal Evaluation

All prepared compounds were tested for antibacterial and antifungal activity using the microdilution broth method. These biological assays were performed on eight bacterial and eight fungal strains of clinical importance. See the Supplementary Material for the experimental details. None of compounds **1**–**18** displayed any antibacterial or antifungal activity up to the highest tested concentration of 500 µM. Propyl esters **1a**–**18a** did not show any antifungal and antibacterial activity, except for compound **11a**. This compound had low antibacterial activity against *Staphylococcus aureus* and *Pseudomonas aeruginosa* with MIC = 250 µM. Only one compound among the methyl esters, **8b**, showed antibacterial activity against *Staphylococcus aureus* with MIC = 250 µM, and the rest of the methyl esters were inactive against the tested bacterial and fungal strains.

### 2.5. Cytotoxicity

The active compounds **16** and **18a** were studied for their in vitro cytotoxic effect in the HepG2 cell line. The results of the experiments are presented as the inhibitory concentration that reduces the viability of the cell population to 50% of the maximal viability, IC_50_. None of the tested compounds (**16**: 4-NO_2_; **18a**: 4-CF_3_) exerted significant cytotoxic effect. Compound **16** showed IC_50_ > 750 µM (precipitation at higher concentration) and ester **18a** showed IC_50_ = 252.4 µM. The values of selectivity index (SI = IC_50_ / MIC) related to *Mtb* were SI > 150 for compound **16** and for **18a** SI = 25.24.

### 2.6. In Silico Docking Study

We performed molecular docking studies on DrpE1 of *Mtb* H37Rv. DprE1 was chosen as a potential new target involved in mycobacterial cell wall synthesis. We studied only acids **1**–**18**, as the methyl and propyl esters are considered as prodrugs which are hydrolyzed in mycobacterium. Molecular Operating Environment (MOE) 2016.08 (Chemical Computing Group, Montreal, QC, Canada) was used to conduct the in silico study. To verify the docking procedure, the originally co-crystalized ligand (quinoxaline) was removed and redocked again with RMSD = 0.24 Å.

PDB structure 4P8N (chain A) was chosen for in silico study of DprE1. The predicted poses of individual ligands **1**–**18** were evaluated with regard to the ligand-receptor interactions and position of original ligand. Eight ligands (**3**, **4**, **6**, **9**, **10**, **11**, **16**, **18**) combining the best docking score, similarity in interactions to the original ligand, and overlapping with the original ligand were considered as the best candidates for DprE1 inhibition. In comparison to the original structure of 2-carboxyquinoxalines, the replacement of -NH-CH_2_- linker by -CONH- group does not radically change the character of binding mode. On the other hand, the loss of the condensed ring along with large CF_3_ substituent seems to decrease the antimycobacterial activity. Probably the large lipophilic substituent is needed for the filling of the hydrophobic binding sub-pocket and will be considered for further investigation. The interactions and binding scores are described and depicted in Supplementary Material.

## 3. Experimental

### 3.1. General Information

All chemicals were of reagent or higher grade of purity and were purchased from Sigma-Aldrich (Steinheim, Germany). The progress of reactions was monitored by Thin Layer Chromatography (TLC; TLC Silica gel 60 F254, Merck, Darmstadt, Germany) with UV detection using a wavelength of 254 nm. Microwave-assisted reactions were performed in a CEM Discover microwave reactor with a focused field (CEM Corporation, Matthews, NC, USA) connected to an Explorer 24 autosampler (CEM Corporation) and this equipment was run under CEM’s Synergy™ software (version 1.38) for setting and monitoring the reaction conditions. The temperature of reactions was monitored by an internal infrared sensor. Propyl esters and methyl esters were purified by preparative flash chromatograph CombiFlash^®^ *R*_f_ (Teledyne Isco Inc., Lincoln, NE, USA). The type of elution was gradient, using the mixture of hexane (LachNer, Neratovice, Czech Republic) and ethyl acetate (Penta, Prague, Czech Republic) as mobile phase. Silica gel (0.040–0.063 nm, Merck) was used as the stationary phase. NMR spectra were taken in DMSO-*d*_6_ with a Varian VNMR S500 spectrometer (499.87 MHz for ^1^H and 125.71 MHz for ^13^C; Varian Corporation, Palo Alto, CA, USA). Chemical shifts were reported in ppm (δ) and were referred indirectly to tetramethylsilane. Infrared spectra were recorded with a FT-IR Nicolet 6700 spectrometer (Thermo Scientific, Waltham, MA, USA) using attenuated total reflectance (ATR) on Ge crystal. Elemental analyses were measured using Micro Cube Elemental Analyzer (Elementar Analysensysteme GmbH, Hanau, Germany). Melting points were assessed by SMP3 Stuart Scientific (Bibby Sterling Ltd., Staffordshire, UK) in an open capillary and are uncorrected. Lipophilicity parameter log *P* was calculated by software CS ChemBioDraw Ultra 14.0 (CambridgeSoft, Cambridge, MA, USA).

### 3.2. Chemistry

#### 3.2.1. General Procedure for the Synthesis of Acids **1**–**18**

Pyrazine-2,3-dicarboxylic acid (4.0 g, 23.8 mmol) was dissolved in acetic anhydride (30 mL). The reaction mixture was refluxed for one hour, and subsequently cooled down to 0 °C in ice bath. The obtained crystals of pyrazine-2,3-dicarboxylic anhydride were filtered off (yield 70%).

Pyrazine-2,3-dicarboxylic anhydride (1.0 g, 6.7 mmol) was dissolved in tetrahydrofuran (40 mL) in an Erlenmeyer flask and the corresponding substituted aniline (6.7 mmol, 1 equiv.) was added in one dose. The reaction mixture was stirred for 1 hour at laboratory temperature. Water (30 mL) was added into the mixture followed by the saturated aqueous solution of NaHCO_3_ until pH 6 to form the corresponding 3-(phenylcarbamoyl)pyrazine-2-carboxylic acid **1**–**18**. Obtained crystals were filtered off and washed with water. The progress of the procedure was monitored by TLC eluted with the system water/butanol/acetic acid 5:4:1.

#### 3.2.2. General Procedure for the Synthesis of Propyl and Methyl Esters

Propyl esters **1a**–**15a**, **17a** and **18a** were prepared using microwave irradiation. Substituted acid **1**–**18** (300 mg), propanol (3 mL) and one drop of H_2_SO_4_ were put to a thick-wall vial for the microwave reactions. Conditions for the synthesis were 120 °C, 20 min, and 50 W in the pressurized vial. The progress of the reactions was monitored by TLC in system propanol/30% aq. sol. of ammonia 3:1. The reaction mixture was adsorbed on silica and purified by flash chromatography using gradient elution with ethyl-acetate (50–90%) in hexane. Methyl esters **1b**, **4b**–**6b**, **8b**, **11b**–**15b** and **17b**, **18b** were prepared using the same procedure as propyl esters using methanol instead as the solvent.

#### 3.2.3. Analytical Data of Prepared Compounds

*3-(Phenylcarbamoyl)pyrazine-2-carboxylic acid* (**1**) [31]. Pale yellow solid. Yield 91%; m.p. 163.1–164.6 °C; IR (cm^−1^): 3341 (N-H, CONH), 3051 (O-H, COOH), 1712 (C=O, COOH), 1682 (C=O, CONH); ^1^H-NMR δ 13.77 (bs, 1H, COOH), 10.75 (s, 1H, NH), 8.92–8.86 (m, 2H, pyr.), 7.80–7.75 (m, 2H, Ar), 7.40–7.34 (m, 2H, Ar), 7.16–7.11 (m, 1H, Ar); ^13^C-NMR δ 166.45, 162.61, 146.40, 145.85, 145.80, 144.69, 138.57, 128.98, 124.39, 120.25; Elemental analysis: calc. for C_12_H_9_N_3_O_3_ (MW 243.22): 59.26% C, 3.73% H, 17.28% N; found 59.34% C, 3.61% H, 17.24% N.

*3-[(2-Hydroxyphenyl)carbamoyl]pyrazine-2-carboxylic acid* (**2**). Yellow solid. Yield 95%; m.p. 266.4–269.3 °C; IR (cm^−1^): 3278 (O-H, OH), 3106 (N-H, CONH), 2854 (O-H, COOH), 1713 (C=O, COOH), 1611 (C=O, CONH); ^1^H-NMR δ 13.74 (bs, 1H, COOH), 10.31 (s, 1H, OH), 10.15 (s, 1H, NH), 8.93 (d, *J* = 2.5 Hz, 1H, pyr.), 8.90 (d, *J* = 2.5 Hz, 1H, pyr.), 8.24–8.20 (m, 1H, Ar), 7.03–6.93 (m, 2H, Ar), 6.88–6.83 (m, 1H, Ar); ^13^C-NMR δ 166.92, 160.10, 147.86, 147.12, 146.85, 144.27, 141.64, 125.81, 125.03, 119.93, 119.48, 115.13; Elemental analysis: calc. for C_12_H_9_N_3_O_4_ (MW 259.22): 55.60% C, 3.50% H, 16.21% N; found 55.77% C, 3.39% H, 16.07% N.

*3-[(4-Methoxyphenyl)carbamoyl]pyrazine-2-carboxylic acid* (**3**). Yellow solid. Yield 98%; m.p. 167.1–168.0 °C; IR (cm^−1^): 3330 (N-H, CONH), 3169 (O-H, COOH), 1757 (C=O, COOH), 1665 (C=O, CONH), 1243 (C-O, OCH_3_); ^1^H-NMR δ 13.74 (bs, 1H, COOH), 10.64 (s, 1H, CONH), 8.89–8.86 (m, 2H, pyr.), 7.73–7.68 (m, 2H, Ar), 6.96–6.92 (m, 2H, Ar), 3.74 (s, 3H, CH_3_); ^13^C-NMR δ 166.56, 161.97, 156.06, 146.67, 145.76, 145.47, 144.51, 131.65, 121.79, 114.08, 55.40; Elemental analysis: calc. for C_13_H_11_N_3_O_4_ (MW 273.25): 57.14% C, 4.06% H, 15.38% N; found 56.68% C, 3.93% H, 15.13% N.

*3-[(2,4-Dimethoxyphenyl)carbamoyl]pyrazine-2-carboxylic acid* (**4**). Yellow solid. Yield 85%; m.p. 199.6–200.3 °C; IR (cm^−1^): 3309 (N-H, CONH), 3129 (O-H, COOH), 1741 (C=O, COOH), 1667 (C=O, CONH); ^1^H-NMR δ 13.73 (bs, 1H, COOH), 9.95 (s, 1H, NH), 8.91 (d, *J* = 2.4 Hz, 1H, pyr.), 8.87 (d, *J* = 2.4 Hz, 1H, pyr.), 8.11 (d, *J* = 8.8 Hz, 1H, Ar), 6.70 (d, *J* = 2.6 Hz, 1H, Ar), 6.58–6.55 (m, 1H, Ar), 3.89 (s, 3H, CH_3_), 3.77 (s, 3H, CH_3_); ^13^C-NMR δ 166.87, 160.06, 157.18, 150.63, 147.69, 146.60, 144.22, 142.07, 121.24, 119.89, 104.50, 99.14, 56.27, 55.56; Elemental analysis: calc. for C_14_H_13_N_3_O_5_ (MW 303.27): 55.45% C, 4.32% H, 13.86% N; found 55.46% C, 4.31% H, 13.84% N.

*3-[(2,5-Dimethylphenyl)carbamoyl]pyrazine-2-carboxylic acid* (**5**). Grey solid. Yield 81%; m.p. 177.4–178.8 °C; IR (cm^−1^): 3339 (N-H, CONH), 2918 (O-H, COOH), 1729(C=O, COOH),1687 (C=O, CONH); ^1^H-NMR δ 13.75 (bs, 1H, COOH), 10.15 (s, 1H, NH), 8.91–8.87 (m, 2H, pyr.), 7.40–7.38 (m, 1H, Ar), 7.14 (d, *J* = 7.7 Hz, 1H, Ar), 6.99–6.95 (m, 1H, Ar), 2.28 (s, 3H, CH_3_), 2.22 (s, 3H, CH_3_); ^13^C-NMR δ 166.53, 162.37, 146.50, 145.90, 145.30, 144.69, 135.48, 135.36, 130.39, 129.06, 126.63, 125.43, 20.80, 17.39; Elemental analysis: calc. for C_14_H_13_N_3_O_3_ (MW 271.28): 61.99% C, 4.83% H, 15.49% N; found 62.46% C, 4.73% H, 15.53% N.

*3-[(4-Ethylphenyl)carbamoyl]pyrazine-2-carboxylic acid* (**6**). Beige solid. Yield 98%; m.p. 159.1–159.9 °C; IR (cm^−1^): 3314 (N-H, CONH), 2972 (O-H, COOH), 1704 (C=O, COOH), 1664 (C=O, CONH); ^1^H-NMR δ 10.68 (s, 1H, NH), 8.90–86 (m, 2H, pyr.), 7.71–7.65 (m, 2H, Ar), 7.23–7.16 (m, 2H, Ar), 2.58 (q, *J* = 7.6 Hz, 2H, CH_2_), 1.17 (t, *J* = 7.6 Hz, 3H, CH_3_); ^13^C-NMR δ 166.50, 162.32, 146.52, 145.79, 145.67, 144.60, 139.87, 136.24, 128.16, 120.32, 27.85, 15.88; Elemental analysis: calc. for C_14_H_13_N_3_O_3_ (MW 271.28): 61.99% C, 4.83% H, 15.49% N; found 61.51% C, 5.24% H, 15.08% N.

*3-[(4-Fluorophenyl)carbamoyl]pyrazine-2-carboxylic acid* (**7**) [27]. Grey solid. Yield 90%; m.p. 168.7–169.6 °C; IR (cm^−1^): 3358 (N-H, CONH), 3069 (O-H, COOH), 1730 (C=O, COOH), 1683 (C=O, CONH); ^1^H-NMR δ 13.79 (bs, 1H, COOH), 10.85 (s, 1H, CONH), 8.92–8.87 (m, 2H, pyr.), 7.84–7.78 (m, 2H, Ar), 7.24–7.18 (m, 2H, Ar); ^13^C-NMR δ 166.46, 162.48, 158.75 (d, *J* = 240.8 Hz), 146.53, 145.93, 145.51, 144.66, 134.95 (d, *J* = 2.5 Hz), 122.16 (d, *J* = 8.0 Hz), 115.59 (d, *J* = 22.3 Hz); Elemental analysis: calc. for C_12_H_8_FN_3_O_3_ (MW 261.21): 55.18% C, 3.09% H, 16.09% N; found 54.96% C, 3.15% H, 15.85% N.

*3-[(2,4-Difluorophenyl)carbamoyl]pyrazine-2-carboxylic acid* (**8**). Grey solid. Yield 94%; m.p. 187.0–188.0 °C; IR (cm^−1^): 3346 (N-H, CONH), 2906 (O-H, COOH), 1702 (C=O, COOH), 1654 (C=O, CONH);^1^H-NMR δ 13.79 (bs, 1H, COOH), 10.54 (s, 1H, NH), 8.91 (d, 1H, *J* = 2.7 Hz, pyr.), 8.89 (d, 1H, *J* = 2.7 Hz, pyr.), 7.86–7.79 (m, 1H, Ar), 7.42–7.35 (m, 1H, Ar), 7.17–7.12 (m, 1H, Ar); ^13^C-NMR δ 165.4, 162.7, 159.5 (dd, *J* = 245.1 Hz, *J* = 11.4 Hz), 155.1 (dd, *J* = 249.9 Hz, *J* = 12.4 Hz), 146.4, 146.2, 144.8, 144.6, 126.7 (dd, *J* = 9.6 Hz, *J* = 2.9 Hz), 122.0 (dd, *J* = 12.4 Hz, *J* = 3.9 Hz), 111.5 (dd, *J* = 21.9 Hz, *J* = 3.8 Hz), 104.6 (dd, *J* = 26.6 Hz, *J* = 23.9 Hz); Elemental analysis: calc. for C_12_H_7_F_2_N_3_O_3_ (MW 279.20): 51.62% C, 2.53% H, 15.05% N; found 51.41% C, 2.05% H, 14.72% N.

*3-[(4-Chlorophenyl)carbamoyl]pyrazine-2-carboxylic acid* (**9**) [32]. White solid. Yield 98%; m.p. 171.1–173.2 °C; IR (cm^−1^): 3331 (N-H, CONH), 2584 (O-H, COOH), 1708 (C=O, COOH), 1692 (C=O, CONH); ^1^H-NMR δ 13.81 (bs, 1H, COOH), 10.92 (s, 1H, CONH), 8.91–8.88 (m, 2H, pyr.), 7.84–7.80 (m, 2H, Ar), 7.45–7.41 (m, 2H, Ar); ^13^C-NMR δ 166.38, 162.70, 146.41, 145.97, 145.52, 144.70, 137.53, 128.90, 128.05, 121.83; Elemental analysis: calc. for C_12_H_8_ClN_3_O_3_ (MW 277.66): 51.91% C, 2.90% H, 15.13% N; found 52.24% C, 2.83% H, 15.25% N.

*3-[(3,4-Dichlorophenyl)carbamoyl]pyrazine-2-carboxylic acid* (**10**). White solid. Yield 85%; m.p. 290.0–292.1 °C; IR (cm^−1^): 3274 (N-H, CONH), 2898 (O-H, COOH), 1724 (C=O, COOH), 1671 (C=O, CONH); ^1^H-NMR δ 11.10 (s, 1H, NH), 8.92 (d, *J* = 2.5 Hz, 1H, pyr.), 8.90 (d, *J* = 2.5 Hz, 1H, pyr.), 8.16 (d, *J* = 2.4 Hz, 1H, Ar), 7.77–7.71 (m, 1H, Ar), 7.63 (d, *J* = 8.8 Hz, 1H, Ar); ^13^C-NMR δ 166.35, 162.94, 146.51, 146.23, 145.03, 144.76, 138.65, 131.26, 130.96, 126.00, 121.49, 120.37; Elemental analysis: calc. for C_12_H_7_Cl_2_N_3_O_3_ (MW 312.11): 46.18% C, 2.26% H, 13.46% N; found 46.18% C, 2.24% H, 13.32% N.

*3-[(4-Bromophenyl)carbamoyl]pyrazine-2-carboxylic acid* (**11**) [33]. White solid. Yield 84%; m.p. 171.1–171.9 °C; IR (cm^−1^): 3312 (N-H, CONH), 2973 (O-H, COOH), 1706 (C=O, COOH), 1662 (C=O, CONH); ^1^H-NMR δ 10.92 (s, 1H, NH), 8.92–8.86 (m, 2H, pyr.), 7.80–7.72 (m, 2H, Ar), 7.59–7.52 (m, 2H, Ar); ^13^C-NMR δ 166.39, 162.75, 146.43, 145.98, 145.55, 144.71, 137.96, 131.82, 122.20, 116.16; Elemental analysis: calc. for C_12_H_8_BrN_3_O_3_ (MW 322.12): 44.75% C, 2.50% H, 13.05% N; found 44.30% C, 2.62% H, 12.55% N.

*3-[(5-Fluoro-2-methylphenyl)carbamoyl]pyrazine-2-carboxylic acid* (**12**). Beige solid. Yield 92%; m.p. 185.0–186.1 °C; IR (cm^−1^): 3301 (N-H, CONH), 3187 (O-H, COOH), 1746 (C=O, COOH), 1670 (C=O, CONH); ^1^H-NMR δ 13.83 (bs, 1H, COOH), 10.26 (s, 1H, NH), 8.92 (d, 1H, *J* = 2.4 Hz, pyr.), 8.90 (d, 1H, *J* = 2.4 Hz, pyr.), 7.51 (dd, 1H, *J* = 9.1 Hz *J* = 2.9 Hz, Ar), 7.01 (dd, 1H, *J* = 9.1 Hz *J* = 6.9 Hz, Ar), 7.00 (dt, 1H, *J* = 9.1 Hz *J =* 2.9 Hz, Ar), 2.25 (s, 3H, CH_3_); ^13^C-NMR δ 166.4, 162.7, 160.4 (d, *J* = 240.3 Hz), 146.3, 146.1, 145.2, 144.9, 136.9 (d, *J* = 10.4 Hz), 131.8 (d, *J* = 8.5 Hz), 127.5 (d, *J =* 2.9 Hz), 112.3 (d, *J* = 21.0 Hz), 111.0 (d, *J* = 24.8 Hz), 17.1; Elemental analysis: calc. for C_13_H_10_FN_3_O_3_ (MW 275.24): 56.73% C, 3.66% H, 15.27% N; found 56.84% C, 3.81% H, 14.93% N.

*3-[(2-Chloro-5-methylphenyl)carbamoyl]pyrazine-2-carboxylic acid* (**13**). Yellow solid. Yield 90%; m.p. 190.1–191.2 °C; IR (cm^−1^): 3342 (N-H, CONH), 2964 (O-H, COOH), 1701 (C=O, COOH), 1664 (C=O, CONH); ^1^H-NMR δ 13.79 (bs, 1H, COOH), 10.34 (s, 1H, NH), 8.94 (d, *J* = 2.5 Hz, 1H, pyr.), 8.91 (d, *J* = 2.5 Hz, 1H, pyr.), 7.91 (d, *J* = 2.0 Hz, 1H, Ar), 7.43 (d, *J* = 8.2 Hz, 1H, Ar), 7.09–7.05 (m, 1H, Ar), 2.33 (s, 3H, CH_3_); ^13^C-NMR δ 166.56, 161.54, 147.20, 146.73, 144.57, 142.86, 137.65, 133.83, 129.34, 127.35, 124.58, 122.67, 20.86; Elemental analysis: calc. for C_13_H_10_ClN_3_O_3_ (MW 291.69): 53.53% C, 3.46% H, 14.41% N; found 53.88% C, 3.37% H, 14.32% N.

*3-[(5-Chloro-2-hydroxyphenyl)carbamoyl]pyrazine-2-carboxylic acid* (**14**). Yellow solid. Yield 97%; m.p. 263.1–265.1 °C; IR (cm^−1^): 3271 (O-H, OH), 3116 (N-H, CONH), 3048 (O-H, COOH), 1709 (C=O, COOH), 1610 (C=O, CONH); ^1^H-NMR δ 13.78 (bs, 1H, COOH), 10.68 (s, 1H, OH), 10.16 (s, 1H, NH), 8.94 (d, *J* = 2.5 Hz, 1H, pyr.), 8.90 (d, *J* = 2.4 Hz, 1H, pyr.), 8.29 (d, *J* = 2.6 Hz, 1H, Ar), 7.07–7.03 (m, 1H, Ar), 6.95 (d, *J* = 8.7 Hz, 1H, Ar); ^13^C-NMR δ 166.74, 160.59, 147.63, 146.97, 146.02, 144.37, 141.58, 126.94, 124.38, 122.70, 119.30, 116.21; Elemental analysis: calc. for C_12_H_8_ClN_3_O_4_ (MW 293.66): 49.08% C, 2.75% H, 14.31% N; found 49.57% C, 2.69% H, 14.17% N.

*3-[(2-Hydroxy-5-nitrophenyl)carbamoyl]pyrazine-2-carboxylic acid* (**15**). Pale yellow solid. Yield 90%; m.p. 290.0–291.3 °C; IR (cm^−1^): 3566 (O-H, OH), 3363 (N-H, CONH), 2972 (O-H, COOH), 1744 (C=O, COOH), 1676 (C=O, CONH), 1545 (N-O, NO_2_), 1349 (N-O, NO_2_); ^1^H-NMR δ 13.73 (bs, 1H, COOH), 12.08 (bs, 1H, OH), 10.25 (s, 1H, NH), 9.16 (d, *J* = 2.8 Hz, 1H, Ar), 8.95 (d, *J* = 2.4 Hz, 1H, pyr.), 8.91 (d, *J* = 2.4 Hz, 1H, pyr.), 8.00–7.96 (m, 1H, Ar), 7.11 (d, *J* = 9.0 Hz, 1H, Ar); ^13^C-NMR δ 166.66, 161.15, 153.61, 147.46, 147.04, 144.49, 141.76, 139.49, 126.02, 121.49, 115.12, 114.78; Elemental analysis: calc. for C_12_H_8_N_4_O_6_ (MW 304.22): 47.38% C, 2.65% H, 18.42% N; found 46.99% C, 3.13% H, 18.15% N.

*3-[(4-Nitrophenyl)carbamoyl]pyrazine-2-carboxylic acid* (**16**) [34]. Yellow solid. Yield 58%; m.p. 224.5–227.2°C; IR (cm^−1^): 3281 (N-H, CONH), 3065 (O-H, COOH), 1713 (C=O, COOH), 1693 (C=O, CONH), 1543 (N-O, NO_2_), 1339 (N-O, NO_2_); ^1^H-NMR δ 13.91 (bs, 1H, COOH), 11.36 (s, 1H, CONH), 8.95–8.92 (m, 2H, pyr.), 8.30–8.25 (m, 2H, Ar), 8.06–8.02 (m, 2H, Ar); ^13^C-NMR δ 166.18, 163.52, 146.24, 146.06, 145.61, 144.96, 144.69, 143.13, 125.12, 120.06; Elemental analysis: calc. for C_12_H_8_N_4_O_5_ (MW 288.22): 50.01% C, 2.80% H, 19.44% N; found 50.28% C, 2.82% H, 19.35% N.

*3-{[3-(Trifluoromethyl)phenyl]carbamoyl}pyrazine-2-carboxylic acid* (**17**). Pale yellow solid. Yield 97%; m.p. 176.3–177.1°C; IR (cm^−1^): 3356 (N-H, CONH), 3064 (O-H, COOH), 1718 (C=O, COOH), 1694 (C=O, CONH); ^1^H-NMR δ 11.14 (s, 1H, NH), 8.93 (d, 1H, *J* = 2.4 Hz, pyr.), 8.91 (d, 1H, *J* = 2.4 Hz, pyr.), 8.30–8.28 (m, 1H, Ar), 8.01 (d, 1H, *J* = 7.9 Hz, Ar), 7.62 (t, 1H, *J* = 7.9 Hz, Ar), 7.50 (d, 1H, *J* = 7.9 Hz, Ar); ^13^C-NMR δ 166.4, 163.1, 146.5, 146.2, 145.2, 144.8, 139.4, 130.3, 129.7 (q, *J* = 31.4 Hz), 124.3 (q, *J* = 271.8 Hz), 123.9, 120.8 (q, *J* = 3.8 Hz), 116.4 (q, *J* = 3.8 Hz); Elemental analysis: calc. for C_13_H_8_F_3_N_3_O_3_ (MW 311.22): 50.17% C, 2.59% H, 13.50% N; found 49.86% C, 2.75% H, 13.42% N.

*3-{[4-(Trifluoromethyl)phenyl]carbamoyl}pyrazine-2-carboxylic acid* (**18**) [35]. White solid. Yield 90%; m.p. 153.5–154.7 °C; IR (cm^−1^): 3306, 1706 (C=O, COOH), 1669 (C=O, CONH); ^1^H-NMR δ 11.13 (s, 1H, NH), 8.92–8.91 (m 1H, pyr.), 8.91–8.89 (m, 1H, pyr.), 8.08–7.96 (m, 2H, Ar), 7.77–7.70 (m, 2H, Ar); ^13^C-NMR δ 166.4, 163.3, 146.4, 146.1, 145.7, 144.8, 142.2, 126.3 (q, *J* = 3.8 Hz), 124.5 (q, *J* = 270.9 Hz), 124.4 (q, *J* = 31.4 Hz), 120.2; Elemental analysis: calc. for C_13_H_8_F_3_N_3_O_3_ (MW 311.22): 50.17% C, 2.59% H, 13.50% N; found 50.42% C, 2.21% H, 13.84% N.

*Propyl 3-(phenylcarbamoyl)pyrazine-2-carboxylate* (**1a**). White solid. Yield 85%; m.p. 74.9–76.5 °C; IR (cm^−1^): 3355 (N-H, CONH), 2961 (C-H), 1734 (C=O, COO), 1686 (C=O, CONH), 1112, 1076 (C-O, COO); ^1^H-NMR δ 10.82 (s, 1H, CONH), 8.94 (s, 2H, pyr.), 7.80–7.77 (m, 2H, Ar), 7.407.35 (m, 2H, Ar), 7.17–7.12 (m, 1H, Ar), 4.26 (t, *J* = 6.6 Hz, 2H, CH_2_), 1.70–1.61 (m, 2H, CH_2_), 0.89 (t, *J* = 7.4 Hz, 3H, CH_3_); ^13^C-NMR δ 165.04, 162.19, 146.14, 145.68, 145.49, 145.11, 138.36, 128.94, 124.47, 120.32, 67.46, 21.45, 10.38; Elemental analysis: calc. for C_15_H_15_N_3_O_3_ (MW 285.30): 63.15% C, 5.30% H, 14.73% N; found 63.41% C, 5.40% H, 14.61% N.

*Propyl 3-[(2-hydroxyphenyl)carbamoyl]pyrazine-2-carboxylate* (**2a**). Pale yellow solid. Yield 64%; m.p. 156.8–159.6 °C; IR (cm^−1^): 3348 (N-H, CONH), 2924 (C-H), 1741 (C=O, COO), 1678 (C=O, CONH), 1111, 1086 (C-O, COO); ^1^H-NMR δ 10.33 (s, 1H, CONH), 10.16 (s, 1H, OH), 8.96 (d, *J* = 2.5 Hz, 1H, pyr.), 8.95 (d, *J* = 2.4 Hz, 1H, pyr.), 8.22–8.17 (m, 1H, Ar), 7.04–6.93 (m, 2H, Ar), 6.89–6.82 (m, 1H, Ar), 4.31 (t, *J* = 6.6 Hz, 2H, CH_2_), 1.76–1.67 (m, 2H, CH_2_), 0.93 (t, *J* = 7.4 Hz, 3H, CH_3_); ^13^C-NMR δ 165.50, 159.83, 147.16, 146.98, 146.63, 144.83, 142.09, 125.63, 125.11, 120.01, 119.45, 115.10, 67.41, 21.49, 10.41; Elemental analysis: calc. for C_15_H_15_N_3_O_4_ (MW 301.30): 59.80% C, 5.02% H, 13.95% N; found 59.83% C, 5.15% H, 13.52% N.

*Propyl 3-[(4-methoxyphenyl)carbamoyl]pyrazine-2-carboxylate* (**3a**). White solid. Yield 43%; m.p. 102.0–102.9 °C; IR (cm^−1^): 3284 (N-H, CONH), 2968 (C-H), 1745 (C=O, COO), 1652 (C=O, CONH), 1159, 1089 (C-O, COO); ^1^H-NMR δ 10.71 (s, 1H, CONH), 8.92 (s, 2H, pyr.), 7.72–7.68 (m, 2H, Ar), 6.96–6.92 (m, 2H, Ar), 4.26 (t, *J* = 6.6 Hz, 2H, CH_2_), 3.75 (s, 3H, CH_3_), 1.70–1.62 (m, 2H, CH_2_), 0.89 (t, *J* = 7.4 Hz, 3H, CH_3_); ^13^C-NMR δ 165.18, 161.58, 156.12, 146.06, 145.74, 145.43, 144.97, 131.44, 121.88, 114.06, 67.40, 55.37, 21.46, 10.39; Elemental analysis: calc. for C_16_H_17_N_3_O_4_ (MW 315.33): 60.94% C, 5.43% H, 13.33% N; found 60.90% C, 5.47% H, 13.21% N.

*Propyl 3-[(2,4-dimethoxyphenyl)carbamoyl]pyrazine-2-carboxylate* (**4a**). Yellow solid. Yield 75%; m.p. 116.4–118.5 °C; IR (cm^−1^): 3371 (N-H, CONH), 2962 (C-H), 1736 (C=O, COO), 1677 (C=O, CONH) ), 112 , 1084 (C-O, COO); ^1^H-NMR δ 9.97 (s, 1H, CONH), 8.76 (d, *J* = 2.4 Hz, 1H, pyr.), 8.68 (d, *J* = 2.4 Hz, 1H, pyr.), 8.43 (d, *J* = 8.6 Hz, 1H, Ar), 6.54–6.50 (m, 2H, Ar), 4.47 (t, *J* = 6.8 Hz, 2H, CH_2_), 3.93 (s, 3H, CH_3_), 3.82 (s, 3H, CH_3_), 1.89–1.80 (m, 2H, CH_2_), 1.03 (t, *J* = 7.5 Hz, 3H, CH_3_); ^13^C-NMR δ 166.02, 158.94, 157.01, 149.92, 148.00, 145.69, 143.06, 142.49, 120.76, 120.42, 103.79, 98.67, 68.05, 55.84, 55.51, 21.77, 10.31; Elemental analysis: calc. for C_17_H_19_N_3_O_5_ (MW 345.36): 59.12% C, 5.55% H, 12.17% N; found 58.70% C, 5.42% H, 11.87% N.

*Propyl 3-[(2,5-dimethylphenyl)carbamoyl]pyrazine-2-carboxylate* (**5a**). White solid. Yield 78%; m.p. 111.8–112.9 °C; IR (cm^−1^): 3354 (N-H, CONH), 2976 (C-H), 1741 (C=O, COO), 1686 (C=O, CONH), 1145, 1079 (C-O, COO); ^1^H-NMR δ 9.54 (s, 1H, CONH), 8.80 (d, *J* = 2.4 Hz, 1H, pyr.), 8.68 (d, *J* = 2.5 Hz, 1H, pyr.), 8.02–8.00 (m, 1H, Ar), 7.11 (d, *J* = 7.6 Hz, 1H, Ar), 6.93 (d, *J* = 7.4 Hz, 1H, Ar), 4.47 (t, *J* = 6.8 Hz, 2H, CH_2_), 2.35 (d, *J* = 6.1 Hz, 6H, CH_3_), 1.90–1.80 (m, 2H, CH_2_), 1.03 (t, *J* = 7.5 Hz, 3H, CH_3_); ^13^C-NMR δ 165.87, 159.33, 148.23, 146.08, 143.00, 142.07, 136.74, 134.76, 130.23, 126.04, 125.13, 122.31, 68.13, 21.76, 21.17, 17.16, 10.29; Elemental analysis: calc. for C_17_H_19_N_3_O_3_ (MW 313.36): 65.16% C, 6.11% H, 13.41% N; found 64.93% C, 5.91% H, 13.47% N.

*Propyl 3-[(4-ethylphenyl)carbamoyl]pyrazine-2-carboxylate* (**6a**). White solid. Yield 80%; m.p. 59.8–61.9 °C; IR (cm^−1^): 3332 (N-H, CONH), 2971 (C-H), 1743 (C=O, COO), 1659 (C=O, CONH), 1154, 1090 (C-O, COO); ^1^H-NMR δ 10.75 (s, 1H, CONH), 8.93 (s, 2H, pyr.), 7.69 (d, *J* = 8.1 Hz, 2H, Ar), 7.20 (d, *J* = 8.1 Hz, 2H, Ar), 4.26 (t, *J* = 6.6 Hz, 2H, CH_2_), 2.58 (q, *J* = 7.6 Hz, 2H, CH_2_), 1.74–1.61 (m, 2H, CH_2_), 1.17 (t, *J* = 7.6 Hz, 3H, CH_3_), 0.89 (t, *J* = 7.4 Hz, 3H, CH_3_); ^13^C-NMR δ 165.11, 161.90, 146.10, 145.62, 145.56, 145.03, 139.97, 136.04, 128.13, 120.41, 67.42, 27.83, 21.46, 15.82, 10.38; Elemental analysis: calc. for C_17_H_19_N_3_O_3_ (MW 313.36): 65.16% C, 6.11% H, 13.41% N; found 64.97% C, 6.02% H, 12.95% N.

*Propyl 3-[(4-fluorophenyl)carbamoyl]pyrazine-2-carboxylate* (**7a**). Beige solid. Yield 72%; m.p. 100.9–103.1 °C; IR (cm^−1^): 3351 (N-H, CONH), 2981 (C-H), 1736 (C=O, COO), 1690 (C=O, CONH), 1159, 1103 (C-O, COO); ^1^H-NMR δ 10.92 (s, 1H, CONH), 8.94 (s, 2H, pyr.), 7.84–7.80 (m, 2H, Ar), 7.24–7.19 (m, 2H, Ar), 4.26 (t, *J* = 6.5 Hz, 2H, CH_2_), 1.70–1.62 (m, 2H, CH_2_), 0.88 (t, *J* = 7.4 Hz, 3H, CH_3_); ^13^C-NMR δ 165.07, 162.08, 158.80 (d, *J* = 241.2 Hz), 146.24, 145.63, 145.37, 145.09, 134.76 (d, *J* = 2.5 Hz), 122.25 (d, *J* = 8.0 Hz), 115.57 (d, *J* = 22.3 Hz), 67.46, 21.46, 10.37; Elemental analysis: calc. for C_15_H_14_FN_3_O_3_ (MW 303.29): 59.40% C, 4.65% H, 13.85% N; found 59.04% C, 4.51% H, 13.59% N.

*Propyl 3-[(2,4-difluorophenyl)carbamoyl]pyrazine-2-carboxylate* (**8a**). White solid. Yield 74%; m.p. 93.3–96.1 °C; IR (cm^−1^): 3358 (N-H, CONH), 2976 (C-H), 1742 (C=O, COO), 1694 (C=O, CONH), 1141, 1099 (C-O, COO); ^1^H-NMR δ 10.61 (bs, 1H, CONH), 8.97–8.93 (m, 2H, pyr.), 7.84–7.77 (m, 1H, Ar), 7.42–7.36 (m, 1H, Ar), 7.18–7.12 (m, 1H, Ar), 4.25 (t, *J* = 6.6 Hz, 2H, CH_2_), 1.71–1.62 (m, 2H, CH_2_), 0.89 (t, *J* = 7.4 Hz, 3H, CH_3_); ^13^C-NMR δ 165.00, 162.29, 159.62 (dd, *J* = 244.9, 11.6 Hz), 155.18 (dd, *J* = 249.7, 12.7 Hz), 146.54, 145.58, 145.24, 144.32, 126.84 (dd, *J* = 9.9, 2.7 Hz), 121.79 (dd, *J* = 12.3, 3.7 Hz), 111.54 (dd, *J* = 22.0, 3.9 Hz), 104.61 (dd, *J* = 27.0, 23.8 Hz), 67.46, 21.45, 10.35.; Elemental analysis: calc. for C_15_H_13_F_2_N_3_O_3_ (MW 321.28): 56.08% C, 4.08% H, 13.08% N; found 55.94% C, 4.12% H, 13.21% N.

*Propyl 3-[(4-chlorophenyl)carbamoyl]pyrazine-2-carboxylate* (**9a**). White solid. Yield 65%; m.p. 121.0–122.1 °C; IR (cm^−1^): 3352 (N-H, CONH), 2981 (C-H), 1737 (C=O, COO), 1690 (C=O, CONH), 1146, 1104 (C-O, COO); ^1^H-NMR δ 10.99 (s, 1H, CONH), 8.94 (s, 2H, pyr.), 7.83 (d, *J* = 8.6 Hz, 2H, Ar), 7.44 (d, *J* = 8.5 Hz, 2H, Ar), 4.26 (t, *J* = 6.5 Hz, 2H, CH_2_), 1.65 (h, *J* = 7.0 Hz, 2H, CH_2_), 0.88 (t, *J* = 7.4 Hz, 3H, CH_3_); ^13^C-NMR δ 164.98, 162.31, 146.29, 145.50, 145.40, 145.15, 137.34, 128.89, 128.18, 121.91, 67.49, 21.45, 10.37; Elemental analysis: calc. for C_15_H_14_ClN_3_O_3_ (MW 319.75): 56.35% C, 4.41% H, 13.14% N; found 56.50% C, 4.39% H, 13.20% N.

*Propyl 3-[(3,4-dichlorophenyl)carbamoyl]pyrazine-2-carboxylate* (**10a**). White solid. Yield 34%; m.p. 101.4–102.2 °C; IR (cm^−1^): 3336 (N-H, CONH), 2975 (C-H), 1735 (C=O, COO), 1697 (C=O, CONH), 1148, 1082 (C-O, COO); ^1^H-NMR δ 11.17 (s, 1H, CONH), 8.97–8.95 (m, 2H, pyr.), 8.16 (d, *J* = 2.5 Hz, 1H, Ar), 7.77 (dd, *J* = 8.8, 2.5 Hz, 1H, Ar), 7.64 (d, *J* = 8.8 Hz, 1H, Ar), 4.27 (t, *J* = 6.5 Hz, 2H, CH_2_), 1.72–1.60 (m, 2H, CH_2_), 0.89 (t, *J* = 7.4 Hz, 3H, CH_3_); ^13^C-NMR δ 164.92, 162.54, 146.52, 145.56, 145.18, 144.91, 138.45, 131.20, 130.93, 126.11, 121.58, 120.43, 67.53, 21.45, 10.36; Elemental analysis: calc. for C_15_H_13_Cl_2_N_3_O_3_ (MW 354.19): 50.87% C, 3.70% H, 11.86% N; found 50.77% C, 3.71% H, 11.49% N.

*Propyl 3-[(4-bromophenyl)carbamoyl]pyrazine-2-carboxylate* (**11a**). White solid. Yield 68%; m.p. 86.6–88.6 °C; IR (cm^−1^): 3337 (N-H, CONH), 2966 (C-H), 1740 (C=O, COO), 1693 (C=O, CONH), 1142, 1083 (C-O, COO); ^1^H-NMR δ 10.99 (s, 1H, CONH), 8.94 (s, 2H, pyr.), 7.79–7.75 (m, 2H, Ar), 7.58–7.54 (m, 2H, Ar.), 4.26 (t, *J* = 6.5 Hz, 2H, CH_2_), 1.70–1.61 (m, 2H, CH_2_), 0.88 (t, *J* = 7.4 Hz, 3H, CH_3_); ^13^C-NMR δ 164.97, 162.33, 146.29, 145.48, 145.41, 145.15, 137.76, 131.80, 122.26, 116.30, 67.49, 21.45, 10.38; Elemental analysis: calc. for C_13_H_14_BrN_3_O_3_ (MW 364.20): 49.47% C, 3.87% H, 11.54% N; found 49.01% C, 3.75% H, 11.18% N.

*Propyl 3-[(5-fluoro-2-methylphenyl)carbamoyl]pyrazine-2-carboxylate* (**12a**). White solid. Yield 33%; m.p. 124.8–127.0 °C; IR (cm^−1^): 3358 (N-H, CONH), 2973 (C-H), 1739 (C=O, COO), 1690 (C=O, CONH), 1135, 1106 (C-O, COO); ^1^H-NMR δ 10.34 (s, 1H, CONH), 8.96 (s, 2H, pyr.), 7.53–7.49 (m, 1H, Ar), 7.33–7.28 (m, 1H, Ar), 7.03–6.98 (m, 1H, Ar), 4.28 (t, *J* = 6.6 Hz, 2H, CH2), 2.24 (s, 3H, CH3), 1.72–1.64 (m, 2H, CH2), 0.91 (t, *J* = 7.4 Hz, 3H, CH3); ^13^C-NMR δ 165.09, 162.07, 160.31 (d, *J* = 240.5 Hz), 146.48, 145.64, 145.20, 144.59, 136.76 (d, *J* = 10.7 Hz), 131.77 (d, *J* = 9.0 Hz), 127.50 (d, *J* = 3.2 Hz), 112.38 (d, *J* = 20.7 Hz), 111.04 (d, *J* = 24.7 Hz), 67.47, 21.49, 17.06, 10.38; Elemental analysis: calc. for C_16_H_16_FN_3_O_3_ (MW 317.32): 60.56% C, 5.08% H, 13.24% N; found 60.14% C, 5.01% H, 12.82% N.

*Propyl 3-[(2-chloro-5-methylphenyl)carbamoyl]pyrazine-2-carboxylate* (**13a**). White solid. Yield 53%; m.p. 110.9–111.4 °C; IR (cm^−1^): 3323 (N-H, CONH), 2974 (C-H), 1736 (C=O, COO), 1697 (C=O, CONH), 1141, 1087 (C-O, COO); ^1^H-NMR δ 10.21 (s, 1H, CONH), 8.82 (d, *J* = 2.4 Hz, 1H, pyr.), 8.73 (d, *J* = 2.4 Hz, 1H, pyr.), 8.42–8.40 (m, 1H, Ar), 7.32–7.26 (m, 1H, Ar), 6.94–6.90 (m, 1H, Ar), 4.49 (t, *J* = 6.8 Hz, 2H, CH_2_), 2.38 (s, 3H, CH_3_), 1.90–1.82 (m, 2H, CH_2_), 1.04 (t, *J* = 7.4 Hz, 3H, CH_3_); ^13^C-NMR δ 165.79, 159.54, 148.23, 146.28, 143.18, 141.68, 138.03, 133.47, 128.72, 126.10, 121.84, 120.47, 68.17, 21.78, 21.28, 10.30; Elemental analysis: calc. for C_16_H_16_ClN_3_O_3_ (MW 333.77): 57.58% C, 4.83% H, 12.59% N; found 57.72% C, 4.87% H, 12.62% N.

*Propyl 3-[(5-chloro-2-hydroxyphenyl)carbamoyl]pyrazine-2-carboxylate* (**14a**). Beige solid. Yield 43%; m.p. 196.6–199.2 °C; IR (cm^−1^): 3369 (N-H, CONH), 2983 (C-H), 1689, 1542, 1339 (N-O, NO_2_), 1156, 1116 (C-O, COO); ^1^H-NMR δ 10.70 (bs, 1H, OH), 10.18 (s, 1H, CONH), 8.97 (d, *J* = 2.4 Hz, 1H, pyr.), 8.95 (d, *J* = 2.5 Hz, 1H, pyr.), 8.25 (d, *J* = 2.7 Hz, 1H, Ar), 7.07–7.04 (m, 1H, Ar), 6.95 (d, *J* = 8.6 Hz, 1H, Ar), 4.32 (t, *J* = 6.6 Hz, 2H, CH_2_), 1.75–1.68 (m, 2H, CH_2_), 0.93 (t, *J* = 7.4 Hz, 3H, CH_3_); ^13^C-NMR δ 165.34, 160.33, 147.13, 146.43, 146.09, 144.94, 141.99, 126.78, 124.50, 122.65, 119.33, 116.22, 67.48, 21.50, 10.40; Elemental analysis: calc. for C_15_H_14_ClN_3_O_4_ (MW 335.74): 53.66% C, 4.20% H, 12.52% N; found 53.24% C, 3.85% H, 12.39% N.

*Propyl 3-[(2-hydroxy-5-nitrophenyl)carbamoyl]pyrazine-2-carboxylate* (**15a**). Beige solid. Yield 58%; m.p. 171.4 °C decomposition; IR (cm^−1^): 3324 (N-H, CONH), 3208 (O-H, OH), 2968 (C-H), 1727 (C=O, COO), 1678 (C=O, CONH), 1094, 1071 (C-O, COO); ^1^H-NMR δ 12.16 (bs, 1H, OH), 10.29 (s, 1H, CONH), 9.12 (d, *J* = 2.9 Hz, 1H, Ar), 8.99 (d, *J* = 2.4 Hz, 1H, pyr.), 8.96 (d, *J* = 2.4 Hz, 1H, pyr.), 8.01–7.97 (m, 1H, Ar), 7.11 (d, *J* = 9.0 Hz, 1H, Ar), 4.33 (t, *J* = 6.6 Hz, 2H, CH_2_), 1.77–1.67 (m, 2H, CH_2_), 0.94 (t, *J* = 7.4 Hz, 3H, CH_3_); ^13^C-NMR δ 165.23, 160.90, 153.75, 147.20, 146.22, 145.06, 142.17, 139.44, 125.87, 121.63, 115.21, 114.81, 67.55, 21.52, 10.41; Elemental analysis: calc. for C_15_H_14_N_4_O_6_ (MW 346.30): 52.03% C, 4.08% H, 16.18% N; found 51.60% C, 4.31% H, 16.60% N.

*Propyl 3-{[3-(trifluoromethyl)phenyl]carbamoyl}pyrazine-2-carboxylate* (**17a**). White solid. Yield 53%; m.p. 80.7–83.3 °C; IR (cm^−1^): 3347 (N-H, CONH), 2974 (C-H), 1745 (C=O, COO), 1697 (C=O, CONH), 1148, 1105 (C-O, COO); ^1^H-NMR δ 9.69 (s, 1H, CONH), 8.82 (d, *J* = 2.4 Hz, 1H, pyr.), 8.68 (d, *J* = 2.4 Hz, 1H, pyr.), 8.02 (d, *J* = 1.9 Hz, 1H, Ar), 7.96–7.93 (m, 1H, Ar), 7.51 (t, *J* = 8.0 Hz, 1H, Ar), 7.45–7.42 (m, 1H, Ar), 4.48 (t, *J* = 6.8 Hz, 2H, CH_2_), 1.90–1.82 (m, 2H, CH_2_), 1.04 (t, *J* = 7.4 Hz, 3H, CH_3_); ^13^C-NMR δ 165.68, 159.76, 148.33, 146.49, 142.98, 141.19, 137.48, 131.55 (q, *J* = 32.7 Hz), 129.70, 123.74 (q, *J* = 272.4 Hz), 122.90, 121.48 (q, *J* = 3.8 Hz), 120.49, 116.62 (q, *J* = 4.1 Hz), 68.27, 21.76, 10.29; Elemental analysis: calc. for C_16_H_14_F_3_N_3_O_3_ (MW 353.30): 54.39% C, 3.99% H, 11.89% N; found 53.95% C, 4.03% H, 11.65% N.

*Propyl 3-{[4-(trifluoromethyl)phenyl]carbamoyl}pyrazine-2-carboxylate* (**18a**). White solid. Yield 60%; m.p. 102.0–102.9 °C; IR (cm^−1^): 3308 (N-H, CONH), 2965 (C-H), 1741 (C=O, COO), 1677 (C=O, CONH), 1110, 1070 (C-O, COO); ^1^H-NMR δ 11.20 (s, 1H, CONH), 8.97 (s, 2H, pyr.), 8.01 (d, *J* = 8.4 Hz, 2H, Ar), 7.75 (d, *J* = 8.6 Hz, 2H, Ar), 4.27 (t, *J* = 6.6 Hz, 2H, CH_2_), 1.69–1.61 (m, 2H, CH_2_), 0.88 (t, *J* = 7.4 Hz, 3H, CH_3_); ^13^C-NMR δ 165.16, 163.12, 146.70, 145.75, 145.62, 145.56, 142.25, 126.56 (q, *J* = 3.8 Hz), 124.78 (q, *J* = 32.1 Hz), 124.72 (q, *J* = 271.4 Hz), 120.61, 67.82, 21.73, 10.63; Elemental analysis: calc. for C_16_H_14_F_3_N_3_O_3_ (MW 353.30): 54.39% C, 3.99% H, 11.89% N; found 54.06% C, 4.05% H, 11.97% N.

*Methyl 3-(phenylcarbamoyl)pyrazine-2-carboxylate* (**1b**). Pale yellow solid. Yield 71%; m.p. 81.8–82.7 °C; IR (cm^−1^): 3349 (N-H, CONH), 2960 (C-H), 1735 (C=O, COO), 1678 (C=O, CONH), 1142, 1087 (C-O, COO); ^1^H-NMR δ 10.83 (s, 1H, NH), 8.96–8.93 (m, 2H, pyr.), 7.81–7.76 (m, 2H, Ar), 7.40–7.35 (m, 2H, Ar), 7.18–7.12 (m, 1H, Ar), 3.89 (s, 3H, CH_3_); ^13^C-NMR δ 165.58, 161.96, 146.65, 146.24, 145.52, 145.15, 138.24, 128.95, 124.56, 120.52, 53.07; Elemental analysis: calc. for C_13_H_11_N_3_O_3_ (MW 257.25): 60.70% C, 4.31% H, 16.33% N; found 60.35% C, 4.55% H, 16.79% N.

*Methyl 3-[(2,4-dimethoxyphenyl)carbamoyl]pyrazine-2-carboxylate* (**4b**). Yellow solid. Yield 59%; m.p. 192.5–194.2 °C; IR (cm^−1^): 3367 (N-H, CONH), 2965 (C-H), 1740 (C=O, COO), 1676 (C=O, CONH), 1124, 1084 (C-O, COO); ^1^H-NMR δ 9.99 (s, 1H, NH), 8.95 (d, *J* = 2.4 Hz, 1H, pyr.), 8.94 (d, *J* = 2.4 Hz, 1H, pyr.), 8.06 (d, *J* = 8.8 Hz, 1H, Ar), 6.71 (d, *J* = 2.6 Hz, 1H, Ar), 6.59–6.55 (m, 1H, Ar), 3.90 (d, *J* = 6.9 Hz, 6H, CH 3), 3.77 (s, 3H, CH 3); ^13^C-NMR δ 165.88, 159.72, 157.33, 150.75, 146.81, 146.26, 144.91, 142.39, 121.52, 119.60, 104.56, 99.12, 56.27, 55.56, 53.00; Elemental analysis: calc. for C_15_H_15_N_3_O_5_ (MW 317.30): 56.78% C, 4.77% H, 13.24% N; found 56.83% C, 4.56% H, 13.44% N.

*Methyl 3-[(2,5-dimethylphenyl)carbamoyl]pyrazine-2-carboxylate* (**5b**). Pale yellow solid. Yield 67%; m.p. 132.1–134.6 °C; IR (cm^−1^): 3379 (N-H, CONH), 2953 (C-H), 1735 (C=O, COO), 1683 (C=O, CONH), 1121, 1082 (C-O, COO); ^1^H-NMR δ 10.26 (s, 1H, NH), 8.96–8.93 (m, 2H, pyr.), 7.38–7.35 (m, 1H, Ar), 7.15 (d, *J* = 7.7 Hz, 1H, Ar), 7.01–6.96 (m, 1H, Ar), 3.88 (s, 3H, CH_3_), 2.29 (s, 3H, CH_3_), 2.21 (s, 3H, CH_3_); ^13^C-NMR δ 165.65, 161.75, 146.33, 145.63, 145.19, 144.69, 135.44, 135.29, 130.39, 129.19, 126.81, 125.52, 53.02, 20.77, 17.34; Elemental analysis: calc. for C_15_H_15_N_3_O_3_ (MW 285.30): 63.15% C, 5.30% H, 14.73% N; found 62.71% C, 5.49% H, 14.92% N.

*Methyl 3-[(4-ethylphenyl)carbamoyl]pyrazine-2-carboxylate* (**6b**). Pale brown solid. Yield 58%; m.p. 89.1–90.7 °C; IR (cm^−1^): 3356 (N-H, CONH), 2962 (C-H), 1737 (C=O, COO), 1686 (C=O, CONH), 1147, 1083 (C-O, COO); ^1^H-NMR δ 10.76 (s, 1H, NH), 8.95–8.92 (m, 2H, pyr.), 7.72–7.67 (m, 2H, Ar), 7.23–7.18 (m, 2H, Ar), 3.88 (s, 3H, CH_3_), 2.58 (q, *J* = 7.6 Hz, 2H, CH_2_), 1.19–1.14 (m, 3H, CH_3_); ^13^C-NMR δ 165.63, 161.69, 146.19, 145.62, 145.12, 145.08, 140.07, 135.92, 128.14, 120.59, 53.04, 27.84, 15.84; Elemental analysis: calc. for C_15_H_15_N_3_O_3_ (MW 285.30): 63.15% C, 5.30% H, 14.73% N; found 62.88% C, 5.23% H, 14.92% N.

*Methyl 3-[(2,4-difluorophenyl)carbamoyl]pyrazine-2-carboxylate* (**8b**). White solid. Yield 72%; m.p. 165.9–167.4 °C; IR (cm^−1^): 3348 (N-H, CONH), 2965 (C-H), 1739 (C=O, COO), 1693 (C=O, CONH), 1143, 1103, 1082 (C-O, COO); ^1^H-NMR δ 10.63 (bs, 1H, NH), 8.96 (s, 2H, pyr.), 7.82–7.74 (m, 1H, Ar), 7.43–7.36 (m, 1H, Ar), 7.18–7.11 (m, 1H, Ar), 3.87 (s, 3H, CH_3_); ^13^C-NMR δ 165.5, 162.2, 159.7 (dd, *J =* 245.1 Hz, *J =* 13.7 Hz), 155.4 (dd, *J =* 249.9 Hz, *J* = 13.5 Hz), 146.7, 145.5, 143.4, 144.1, 127.2 (dd, *J =* 9.6 Hz, *J =* 1.9 Hz), 121.7 (dd, *J* = 12.4 Hz, *J* = 3.9 Hz), 111.6 (dd, *J* = 22.0 Hz, *J* = 3.9 Hz), 104.7 (dd, *J =* 26.6 Hz, *J =* 23.8 Hz), 53.1; Elemental analysis: calc. for C_13_H_9_F_2_N_3_O_3_ (MW 293.23): 53.25% C, 3.09% H, 14.33% N; found 53.36% C, 2.76% H, 14.53% N.

*Methyl 3-[(4-bromophenyl)carbamoyl]pyrazine-2-carboxylate* (**11b**). White solid. Yield 66%; m.p. 128.5–130.4 °C; IR (cm^−1^): 3326 (N-H, CONH), 2956 (C-H), 1742 (C=O, COO), 1686 (C=O, CONH), 1174, 1071 (C-O, COO); ^1^H-NMR δ 10.99 (s, 1H, NH), 8.96–8.94 (m, 2H, pyr.), 7.80–7.76 (m, 2H, Ar), 7.58–7.55 (m, 2H, Ar), 3.88 (s, 3H, CH 3); ^13^C-NMR δ 165.51, 162.07, 146.64, 146.39, 145.18, 144.87, 137.64, 131.79, 122.45, 116.39, 53.10; Elemental analysis: calc. for C_13_H_10_BrN_3_O_3_ (MW 336.15): 46.45% C, 3.00% H, 12.50% N; found 46.11% C, 2.76% H, 12.19% N.

*Methyl 3-[(5-fluoro-2-methylphenyl)carbamoyl]pyrazine-2-carboxylate* (**12b**). White solid. Yield 76%; m.p. 174.2–176.9 °C; IR (cm^−1^): 3375 (N-H, CONH), 2955 (C-H), 1741 (C=O, COO), 1692 (C=O, CONH), 1142, 1112 (C-O, COO); ^1^H-NMR δ 10.36 (bs, 1H, NH), 8.97–8.95 (m, 2H, pyr.), 7.49 (dd, 1H, *J* = 9.7 Hz, *J* = 2.9 Hz, Ar), 7.33–7.28 (1H, m, Ar), 7.01 (dt, 1H, *J =* 9.7 Hz *J =* 2.9 Hz, Ar), 3.89 (s, 3H, CH_3_), 2.24 (s, 3H, CH_3_); ^13^C-NMR δ 165.5, 162.0, 160.3 (d, *J =* 240.3 Hz), 146.5, 145.3, 144.5, 144.1, 136.7 (d, *J =* 10.4 Hz), 131.8 (d, *J =* 9.4 Hz), 127.7 (d, *J =* 2.9 Hz), 112.5 (d, *J =* 21.0 Hz), 111.2 (d, *J =* 24.8 Hz), 53.1, 17.0; Elemental analysis: calc. for C_14_H_12_FN_3_O_3_ (MW 289.27): 58.13% C, 4.18% H, 14.53% N; found 57.74% C, 3.97% H, 14.26% N.

*Methyl 3-[(2-chloro-5-methylphenyl)carbamoyl]pyrazine-2-carboxylate* (**13b**). Pale yellow solid. Yield 74%; m.p. 166.7–168.8 °C; IR (cm^−1^): 3349 (N-H, CONH), 2956 (C-H), 1737 (C=O, COO), 1689 (C=O, CONH), 1144, 1088 (C-O, COO); ^1^H-NMR δ 10.43 (s, 1H, NH), 8.99–8.97 (m, 2H, pyr.), 7.86 (d, *J* = 2.0 Hz, 1H, Ar), 7.44 (d, *J* = 8.1 Hz, 1H, Ar), 7.12–7.05 (m, 1H, Ar), 3.91 (s, 3H, CH_3_), 2.33 (s, 3H, CH_3_); ^13^C-NMR δ 165.63, 161.15, 147.02, 146.00, 145.19, 142.85, 137.73, 133.66, 129.36, 127.60, 124.87, 123.02, 53.13, 20.83; Elemental analysis: calc. for C_14_H_12_ClN_3_O_3_ (MW 305.72): 55.00% C, 3.96% H, 13.75% N; found 55.43% C, 3.85% H, 13.88% N.

*Methyl 3-[(5-chloro-2-hydroxyphenyl)carbamoyl]pyrazine-2-carboxylate* (**14b**). Yellow solid. Yield 72%; m.p. 174.2–176.9 °C; IR (cm^−1^): 3316 (N-H, CONH), 2963 (C-H), 1735 (C=O, COO), 1681 (C=O, CONH), 1148, 1091 (C-O, COO); ^1^H-NMR δ 10.71 (s, 1H, OH), 10.19 (s, 1H, NH), 8.98 (d, *J* = 2.4 Hz, 1H, pyr.), 8.96 (d, *J* = 2.5 Hz, 1H, pyr.), 8.25 (d, *J* = 2.5 Hz, 1H, Ar), 7.09–7.03 (m, 1H, Ar), 6.95 (d, 1H, Ar), 3.92 (d, *J* = 9.0 Hz, 3H, CH_3_); ^13^C-NMR δ 165.80, 160.17, 147.20, 146.66, 146.10, 145.02, 141.77, 126.72, 124.55, 122.69, 119.34, 116.23, 53.13; Elemental analysis: calc. for C_13_H_10_ClN_3_O_4_ (MW 307.69): 50.75% C, 3.28% H, 13.66% N; found 50.39% C, 3.19% H, 13.72% N.

*Methyl 3-[(2-hydroxy-5-nitrophenyl)carbamoyl]pyrazine-2-carboxylate* (**15b**). Pale yellow solid. Yield 60%; m.p. 226.4–228.6 °C; IR (cm^−1^): 3317 (N-H, CONH), 2969 (C-H), 1732 (C=O, COO), 1683 (C=O, CONH), 1267, 1095 (C-O, COO); ^1^H-NMR δ 12.10 (bs, 1H, OH), 10.29 (s, 1H, NH), 9.10 (d, *J* = 2.9 Hz, 1H, Ar), 8.99 (d, *J* = 2.4 Hz, 1H, pyr.), 8.97 (d, *J* = 2.4 Hz, 1H, pyr.), 8.01–7.94 (m, 1H, Ar), 7.10 (d, *J* = 9.0 Hz, 1H, Ar), 3.95 (s, 3H, CH_3_); ^13^C-NMR δ 165.71, 160.66, 153.69, 147.28, 146.16, 145.12, 141.83, 139.46, 125.81, 121.65, 115.15, 114.79, 53.19; Elemental analysis: calc. for C_13_H_10_N_4_O_6_ (MW 318.25): 49.06% C, 3.17% H, 17.61% N; found 48.76% C, 3.05% H, 17.52% N.

*Methyl 3-{[3-(trifluoromethyl)phenyl]carbamoyl}pyrazine-2-carboxylate* (**17b**). Yellow solid. Yield 52%; m.p. 106.3–108.5 °C; IR (cm^−1^): 3357 (N-H, CONH), 2919 (C-H), 1748 (C=O, COO), 1693 (C=O, CONH), 1110, 1081 (C-O, COO); ^1^H-NMR δ 9.71 (bs, 1H, NH), 8.82 (d, 1H, *J =* 2.2 Hz, pyr.), 8.69 (d, 1H, *J =* 2.2 Hz, pyr.), 8.08 (s, 1H, Ar), 7.93 (d, 1H, *J =* 8.0 Hz, Ar), 7.51 (t, 1H, *J =* 8.0 Hz, Ar), 7.43 (d, 1H, *J =* 8.0 Hz, Ar), 4.10 (s, 3H, CH_3_); ^13^C-NMR δ 166.0, 159.8, 148.0, 146.5, 145.5, 143.1, 137.4, 131.6 (q, *J =* 33.3 Hz), 129.7, 123.0, 123.7 (q, *J =* 271.9 Hz), 121.6 (q, *J =* 3.8 Hz), 116.7 (q, *J =* 3.9 Hz), 53.4.

*Methyl 3-{[4-(trifluoromethyl)phenyl]carbamoyl}pyrazine-2-carboxylate* (**18b**). White solid. Yield 50%; m.p. 146.8–148.2 °C; IR (cm^−1^): 3297 (N-H, CONH), 2961 (C-H), 1746 (C=O, COO), 1691 (C=O, CONH), 1112, 1063 (C-O, COO); ^1^H-NMR δ 9.73 (bs, 1H, NH), 8.82 (d, 1H, *J =* 2.2 Hz, pyr.), 8.69 (d, 1H, *J =* 2.2 Hz, pyr.), 7.87–7.84 (m, 2H, Ar), 7.65–7.62 (m, 2H, Ar), 4.09 (s, 3H, CH_3_); ^13^C-NMR δ 166.0, 159.8, 148.0, 146.5, 145.5, 143.1, 141.2, 126.8 (q, *J =* 33.8 Hz), 126.4 (q, *J =* 3.8 Hz), 123.9 (q, *J =* 270.9 Hz), 119.7, 53.4; Elemental analysis: calc. for C_14_H_10_F_3_N_3_O_3_ (MW 325.25): 51.70% C, 3.10% H, 12.92% N; found 51.95% C, 3.41% H, 12.83% N.

## 4. Conclusions

In this research project, we focused on the synthesis of 18 POA derivatives with increased lipophilicity achieved by substitution with phenylcarbamoyl moiety in position 3 of the pyrazine ring. Furthermore, we prepared 17 propyl esters and 12 methyl esters as prodrugs to further increase the lipophilicity and enhance the penetration of these derivatives through the mycobacterial cell wall. The recently uncovered antimycobacterial target, DprE1, was evaluated as potential target for our new series of POA derivatives, on the basis of structural similarities with previously published 2-carboxyquinoxalines (Figure 2). The results from a molecular docking study to DprE1 showed that the alteration of -NH-CH_2_- linker of the original scaffold to -CONH- group (title compounds of this study) did not alter the position of the scaffold in DprE1.

Prepared compounds were tested for in vitro growth inhibition activity against *M. tuberculosis* H37Rv and three nontuberculous strains. Five compounds showed moderate activity against *Mtb* (MIC = 50 or 100 μg·mL^−1^) and compound **16** exerted high efficiency with MIC = 1.56 μg·mL^−1^. The whole cell activity of **16** (MIC = 5.0 μM) was fully comparable with the activity of the best template 2-carboxyquinoxalines (Figure 1, MIC = 3.1 μM). With respect to our docking and biological results and the activities of 2-carboxyquinoxalines published previously (Figure 2), a bulky lipophilic substituent on the pyrazine ring, complimentary to the lipophilic cavity, is suggested to be important for the inhibition of DprE1. The lowered antimycobacterial activity of our compounds in whole cell assay could be caused by insufficient penetration through the cell wall (insufficient lipophilicity), low intrinsic affinity to the enzyme, or both. The most lipophilic compound from our series was propyl ester **10a** with log *P* = 2.77, which is significantly lower in comparison with the model 2-carboxyquinoxaline derivative (log *P* = 4.05).

The increase of lipophilicity by the conversion to propyl and methyl esters did not produce any improvement in biological activity in general. Only propyl ester **18a** showed antimycobacterial activity against *Mtb* with MIC = 3.13 μg·mL^−1^, a value similar to its parent acid **18**. Antibacterial and antifungal assays did not reveal any compound with significant activity. As next research, we will focus on the design of compounds with lipophilic/bulky substituents at C-5 or C-6 of the pyrazine core as an attempt to fill in the lipophilic cavity of DprE1.

## Figures and Tables

**Table 1:** Prepared compounds with lipophilicity parameter log *P* and antimycobacterial activity against *Mtb* in μg·mL^−1^ and in μM in brackets.

R	Cpd.	Log *P*	MIC *Mtb* (μg·mL^−1^)	Cpd.	Log *P*	MIC *Mtb* (μg·mL^−1^)	Cpd.	Log *P*	MIC *Mtb* (μg·mL^−1^)
H	1	0.57	>100	1a	1.66	>100	1b	0.83	>100
2-OH	2	0.18	50 (193)	2a	1.27	50 (160)	-	-	-
4-OCH_3_	3	0.44	>100	3a	1.53	>100	-	-	-
2,4-diOCH_3_	4	0.32	>100	4a	1.40	>100	4b	0.58	>100
2,5-diCH_3_	5	1.54	>100	5a	2.63	>100	5b	1.81	>100
4-CH_2_CH_3_	6	1.47	>100	6a	2.56	>100	6b	1.74	>100
4-F	7	0.73	>100	7a	1.81	>100	-	-	-
2,4-diF	8	0.89	>100	8a	1.97	>100	8b	1.15	>100
4-Cl	9	1.13	>100	9a	2.21	>100	-	-	-
3,4-diCl	10	1.69	100	10a	2.77	>100	-	-	-
4-Br	11	1.40	50 (155)	11a	2.49	>100	11b	1.66	>100
5-F-2-CH_3_	12	1.21	>100	12a	2.30	>100	12b	1.48	>100
2-Cl-5-CH_3_	13	1.61	>100	13a	2.70	>100	13b	1.88	>100
5-Cl-2-OH	14	0.74	100	14a	1.83	>100	14b	1.00	>100
2-OH-5-NO_2_	15	−0.26	>100	15a	0.96	>100	15b	0.21	>100
4-NO_2_	16	0.13	1.56 (5)	-	-	-	-	-	-
3-CF_3_	17	1.49	100	17a	2.58	>100	17b	1.75	>100
4-CF_3_	18	1.49	>100	18a	2.58	3.13 (10)	18b	1.75	>100
-	PZA	−1.31	100	POA	−0.66	100	INH	−0.64	0.1–0.2 (0.7–1.5)

INH—isoniazid; PZA—pyrazinamide.

## Supplementary Materials

Supplementary Materials are available online. Docking study and methodologies of biological assays.
