# Supplementary material for: Design, Synthesis, Antimycobacterial Evaluation, and In Silico Studies of 3-(Phenylcarbamoyl)-pyrazine-2-carboxylic Acids
Source: Molecules. 2017 Sep 7;22(9):1491. doi: 10.3390/molecules22091491 (PMC6151461; doi:10.3390/molecules22091491)
Supplement: Supplementary file 1 [file molecules-22-01491-s001.pdf]

Design, Synthesis, Antimycobacterial Evaluation, and *In Silico* Studies of 3-(Phenylcarbamoyl)pyrazine-2-carboxylic Acids

Lucia Semelková [1,*], Petra Janošcová [1], Carlos Fernandes [1], Ghada Bouz [1], Ondřej Janďourek [1], Klára Konečná [1], Pavla Paterová [2], Lucie Navrátilová [1], Jiří Kuneš [1], Martin Doležal [1] and Jan Zitko [1,*]

[1] Faculty of Pharmacy in Hradec Králové, Charles University, Heyrovského 1203, Hradec Králové 500 05, Czech Republic; Petra.Janoscova@seznam.cz (P.J.); oaksdonsad@gmail.com (C.F.); bouzg@faf.cuni.cz (G.B.); JANDO6AA@faf.cuni.cz (O.J.); konecna@faf.cuni.cz (K.K.); navratl2@faf.cuni.cz (L.N.); kunes@faf.cuni.cz (J.K.); dolezalm@faf.cuni.cz (M.D.)

[2] Department of Clinical Microbiology, University Hospital, Sokolská 581, Hradec Králové 500 05, Czech Republic; pavla.paterova@fnhk.cz

* Correspondence: semelkol@faf.cuni.cz (L.S.); jan.zitko@faf.cuni.cz (J.Z.); Tel.: +420-495-067-275 (L.S.)

Supplementary Material

1. In Silico Docking Study

We performed molecular docking studies on DrpE1 of *M. tuberculosis* H37Rv. DprE1 was chosen as a potential new target involved in mycobacterial cell wall synthesis. We studied only acids **1–18**, methyl and propyl esters are considered as prodrugs, which are hydrolyzed in mycobacterium. Molecular Operating Environment (MOE) 2016.08 was used to conduct the *in silico* study. To verify the docking procedure, the originally co-crystalized ligand was removed and redocked again with RMSD = 0.24 Å.

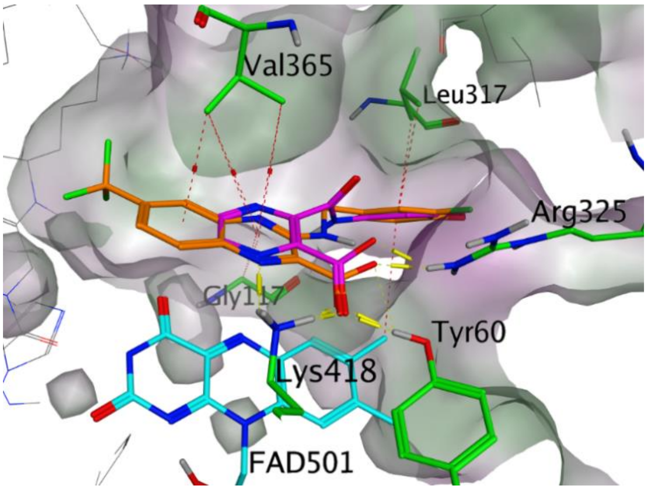

Figure S1. Predicted binding mode of **11** (magenta) in comparison with original ligand (orange).

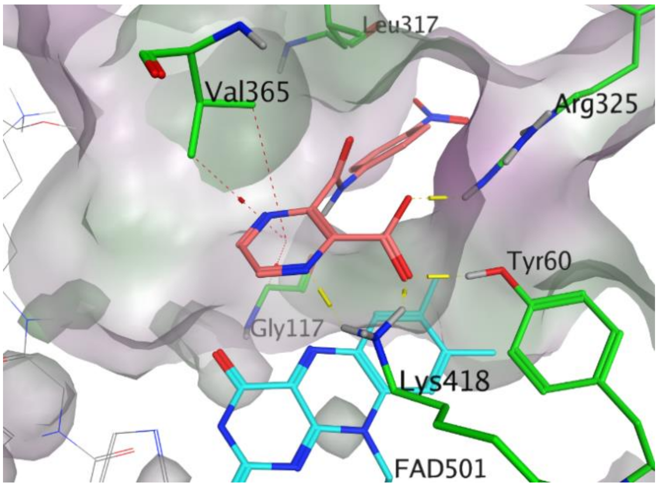

Figure S2. Predicted binding mode of **16** (pink), the most active compound against *Mtb*.

PDB structure 4P8N (chain A) was chosen for *in silico* study of DprE1. The predicted poses of individual ligands **1–18** were evaluated with regard to the ligand-receptor interactions and position of original ligand. Eight ligands (**3**, **4**, **6**, **9**, **10**, **11**, **16**, **18**) combining the best docking score, similarity in interactions to the original ligand, and overlapping with the original ligand were considered as the best candidates for DprE1 inhibition. These compounds form interactions between the carbonyl of the carboxylic group (COOH) and side-chain of Lys418 and Tyr60; the 'hydroxyl' oxygen of COOH with side-chain of Arg325; and N-1 of the pyrazine core creates an interaction with Lys418. Pyrazine ring is stabilized by H-π interactions with Val365 and with Gly117 in a similar way to the original ligand. The original ligand had docking score -8.73 kcal/mol, the best studied compound **4** (R = 2,4-diOCH_3) had docking score -7.28 kcal/mol. When we calculated ligand efficiency (LE = docking score/number of heavy atoms), compound **4** had better LE = -0.33, compared to original ligand´s LE = -0.31. The best compound according to LE was **11** (R = 4-Br) with LE = -0.36. This compound also exhibited π–π interaction with FAD cofactor as the original ligand, see Figure S1. Compound **16**, the most active compound in the whole cell assay, had the docking score -6.96 kcal/mol and LE = -0.33 (Figure S2). In comparison to the original structure of 2-carboxyquinoxalines, the replacement of -NH-CH_2- linker by -CONH- group does not radically change the character of binding mode. On the other hand, the loss of the condensed ring along with large CF_3 substituent seems to decrease antimycobacterial activity. Probably the large lipophilic substituent is needed for the filling of the hydrophobic sub-pocket.

2. Experimental

2.1. Evaluation of In Vitro Antimycobacterial Activity

A microdilution panel method was used. Tested strains *Mycobacterium tuberculosis* H37Rv CNCTC My 331/88 (ATCC 27294), *M. kansasii* CNCTC My 235/80 (ATCC12478) and *M. avium* ssp. *avium* CNCTC My 80/72 (ATCC 15769) were obtained from the Czech National Collection of Type Cultures (CNCTC), National Institute of Public Health (Prague, Czech Republic). Middlebrook 7H9 broth (Sigma-Aldrich, Steinheim, Germany) enriched with the 0.4% of glycerol (Sigma-Aldrich) and 10% of OADC supplement (oleic acid, albumin, dextrose, catalase; Himedia, Mumbai, India) of declared pH = 6.6 was used for cultivation. Tested compounds were dissolved and diluted in DMSO and mixed with broth (25 μL of DMSO solution in 4.475 mL of broth) and placed (100 μL) into microplate wells. Mycobacterial inocula were suspended in isotonic saline solution and the density was adjusted to 0.5–1.0 McFarland. These suspensions were diluted by 10^{-1} and used to inoculate the testing wells, adding 100 μL of suspension to 100 μL of the DMSO/broth solution of tested compound. Final concentrations of tested compounds in wells were 100, 50, 25, 12.5, 6.25, 3.13 and 1.56 μg·mL^{-1}. Isoniazid (INH) was used as positive control (inhibition of growth). Negative control consisted of broth plus DMSO. A total of 30 μL of Alamar Blue working solution (1:1 mixture of 0.1% resazurin sodium salt (aq. sol.) and 10% Tween 80) was added after five days of incubation. Results were then determined after 24 h of incubation. The minimum inhibitory concentration (MIC; μg·mL^{-1}) was determined as the lowest concentration that prevented the blue to

pink color change. MIC values of INH were 6.25–12.5 μg·mL^{-1} against *M. avium*, 3.13–12.5 μg·mL^{-1} against *M. kansasii*, and 0.1–0.2 μg·mL^{-1} against *M. tbc*.

2.2. Antimycobacterial In Vitro Activity Screening Against Mycobacterium smegmatis

The antimycobacterial assay was performed with fast growing *Mycobacterium smegmatis* CCM 4622 (ATCC 607) from the Czech Collection of Microorganisms (Brno, Czech Republic). The technique used for activity determination was microdilution broth panel method using 96-well microtitration plates. The culturing medium was Middlebrook 7H9 (MB) broth (Sigma-Aldrich), enriched with 0.4% of glycerol (Sigma-Aldrich, Steinheim, Germany) and 10% of Middlebrook OADC growth supplement (Himedia). Tested compounds were dissolved in DMSO (Sigma-Aldrich), and the MB broth was then added to achieve a concentration of 2000 μg·mL^{-1}. Standards used for activity determination were INH, rifampicin (RIF), and ciprofloxacin (CPX) (Sigma-Aldrich). Final concentrations were reached by binary dilution followed by the addition of mycobacterial suspension, and were set as 500, 250, 125, 62.5, 31.25, 15.625, 7.81, and 3.91 μg·mL^{-1}, except for the standards of ciprofloxacin and rifampicin, where the final concentrations were 12.5, 6.25, 3.125, 1.56, 0.78, 0.39, 0.195, and 0.098 μg·mL^{-1}. The final concentration of DMSO did not exceeded 2.5% (*v*/*v*) and did not affect the growth of *M. smegmatis*. Plates were also sealed with polyester adhesive film and incubated in the dark at 37 °C, without agitation. The addition of 0.01% solution of resazurin sodium salt followed after 48 h. This stain was prepared by dissolving resazurin sodium salt (Sigma-Aldrich) in deionised water, producing a 0.02% solution. Then, a 10% aqueous solution of Tween 80 (Sigma-Aldrich) was prepared. Both liquids were mixed up making use of the same volumes and filtered through a syringe membrane filter. Microtitration panels were then further incubated for 4 h. Antimycobacterial activity was expressed as the minimal inhibition concentration (MIC) and the value was read on the basis of stain color change (blue color—active compound; pink color—not active compound). The MIC values for the standards were in the range of 7.81–15.625 μg·mL^{-1} for INH, 0.78–1.56 μg·mL^{-1} for RIF, and 0.098–0.195 μg·mL^{-1} for CPX. All experiments were conducted in duplicate.

2.3. Evaluation of In Vitro Antibacterial Activity

Microdilution broth method was used. Antibacterial evaluation was performed against eight bacterial strains from the Czech Collection of Microorganisms (*Staphylococcus aureus* CCM 4516/08, *Escherichia coli* CCM 4517, *Pseudomonas aeruginosa* CCM 1961) or clinical isolates from the Department of Clinical Microbiology, University Hospital and Faculty of Medicine in Hradec Králové, Charles University in Prague, Czech Republic (*Staphylococcus aureus* H 5996/08-methicilin resistant (MRSA), *Staphylococcus epidermidis* H 6966/08, *Enterococcus* sp. J 14365/08, *Klebsiella pneumoniae* D 11750/08, *Klebsiella pneumoniae* J 14368/08-ESBL positive). All strains were subcultured on Mueller-Hinton agar (MHA) (Difco/Becton Dickinson, Detroit, MI, USA) at 35 °C and maintained on the same medium at 4 °C. The compounds were dissolved in DMSO, and the antibacterial activity was determined in Mueller-Hinton liquid broth (Difco/Becton Dickinson), and buffered to pH 7.0. Controls consisted of medium and DMSO alone. The final concentration of DMSO in the test medium did not exceed 1% (*v*/*v*) of the total solution composition. The minimum inhibitory concentration (MIC), defined as the minimum concentration to prevent the visible growth compared to control, was determined after 24 and 48 h of static incubation at 35 °C. The standards were neomycin, bacitracin, penicillin G, ciprofloxacin, and phenoxymethylpenicillin.

2.4. Evaluation of In Vitro Antifungal Activity

Antifungal evaluation was performed using a microdilution broth method against eight fungal strains (*Candida albicans* ATCC 44859, *C. tropicalis* 156, *C. krusei* E28, *C. glabrata* 20/I, *Trichosporon asahii* 1188, *Aspergillus fumigatus* 231, *Lichtheimia corymbifera* 272 and *Trichophyton mentagrophytes* 445). Compounds were dissolved in DMSO and diluted in a twofold manner with RPMI 1640 medium, with glutamine buffered to pH 7.0 (3-morpholinopropane-1-sulfonic acid). The final concentration of DMSO in the tested medium did not exceed 2.5% (*v*/*v*) of the total solution composition. Static incubation was performed in the dark and humidity, at 35 °C, for 24 and 48 h (72 and 120 h for *Trichophyton mentagrophytes*). Drug-free controls were included. The standards were amphotericin B, voriconazole, nystatin, and fluconazole.

2.5. *Cytotoxicity Assays*

The human liver hepatocellular carcinoma cell line HepG2 (passage 12–13), purchased from Health Protection Agency Culture Collections (ECACC, Salisbury, UK), was routinely cultured in Minimum Essential Eagle Medium MEM (Sigma-Aldrich) supplemented with 10% fetal bovine serum (PAA Laboratories GmbH, Pasching, Autria), 2 mM L-glutamine solution (Sigma-Aldrich), and 1% non-essential amino acid solution (Sigma-Aldrich), in a humidified atmosphere containing 5% CO_2 at 37 °C.

For subculturing, the cells were harvested after trypsin/EDTA (Sigma-Aldrich) treatment at 37 °C. To evaluate the cytotoxicity, the HepG2 cells treated with the tested substances were used as experimental groups, whereas untreated HepG2 cells served as control groups.

The HepG2 cells were seeded in a density of 1×10^4 cells per well on a 96-well plate. The following day (24 h after seeding), they were treated with tested substances dissolved in DMSO (maximal incubation concentration of DMSO was 1%). The tested substances were prepared according to their solubility in DMSO, at incubation concentrations of 1–750 μM. The treatment was carried out in a humidified atmosphere containing 5% CO_2 at 37 °C, in triplicate, for 24 h. The controls representing 100% cell viability, 0% cell viability (the cells treated with 10% DMSO), no-cell controls, and vehiculum controls were incubated in triplicate, simultaneously. After 24 h exposure, the reagent from the kit CellTiter 96® Aqueous One Solution Cell Proliferation Assay (Promega, Madison, Wisconsin, USA) was added, according to the recommendation by the manufacturer. After 2 h incubation at 37 °C in humidified, 5% CO_2 atmosphere, the absorbance was recorded at 490 nm. Inhibitory curves were constructed for each compound, plotting incubation concentrations vs. percentage of absorbance relative to untreated control. The standard toxicological parameter IC_{50} was calculated by a nonlinear regression analysis of the inhibitory curves using GraphPad Prism software (version 7, GraphPad Software, Inc., La Jolla, CA, USA).

2.6. *In Silico Docking Study*

In silico experiments were performed in Molecular Operating Environment (MOE) 2016.08 (Chemical Computing Group Inc., Montreal, QC, Canada) using the Amber10:EHT forcefield. DprE1 protein was downloaded from PDB database as entry 4P8N. The protein was prepared by MOE QuickPrep functionality with default settings, which included corrections of structural errors, addition of hydrogens, calculation of partial charges, 3D optimization of *H*-bond network (Protonate3D), deletion of water molecules further than 4.5 Å from ligand or protein, and a restrained minimization of ligand and pocket residues within 8 Å from the ligand. According to the experiments published in [23], we have observed that no molecule of water is important for the protein-ligand or ligand-cofactor interactions, so we removed all solvent molecules. FAD cofactor was defined as a part of the protein.

All compounds were created by MOE Builder and converted to a small library. The library was adjusted for *in silico* docking. Partial charges were computed and compounds were minimized by conjugate gradient method to RMS gradient of 0.1 $kcal \cdot mol^{-1} \cdot Å^{-1}$.

Docking was focused on the pocket defined as residues within 5 Å from the co-crystalized ligand. Parameters of the MOE docking protocol setup for DprE1: Docking stage – Placement method: Triangle Matcher; Score: London dG; retain 30 poses. Refinement stage – Rigid receptor; Score: GBVI/WSA dG; retain 5 poses. Ligand conformations – Conformation import.
